# TDP43 nuclear export and neurodegeneration in models of amyotrophic lateral sclerosis and frontotemporal dementia

**DOI:** 10.1038/s41598-018-22858-w

**Published:** 2018-03-15

**Authors:** Hilary C. Archbold, Kasey L. Jackson, Ayush Arora, Kaitlin Weskamp, Elizabeth M.-H. Tank, Xingli Li, Roberto Miguez, Robert D. Dayton, Sharon Tamir, Ronald L. Klein, Sami J. Barmada

**Affiliations:** 1 Department of Neurology, University of Michigan, Ann Arbor, MI USA; 2 Department of Pharmacology, Toxicology & Neuroscience, Louisiana State University Health Sciences Center, Shreveport, LA USA; 3 Neuroscience Graduate Program, University of Michigan, Ann Arbor, MI USA; 4 Karyopharm Therapeutics, Newton, MA USA; 5 Cellular & Molecular Biology Program, University of Michigan, Ann Arbor, MI USA

## Abstract

Amyotrophic lateral sclerosis (ALS) and frontotemporal dementia (FTD) are progressive neurodegenerative disorders marked in most cases by the nuclear exclusion and cytoplasmic deposition of the RNA binding protein TDP43. We previously demonstrated that ALS–associated mutant TDP43 accumulates within the cytoplasm, and that TDP43 mislocalization predicts neurodegeneration. Here, we sought to prevent neurodegeneration in ALS/FTD models using selective inhibitor of nuclear export (SINE) compounds that target exportin-1 (XPO1). SINE compounds modestly extend cellular survival in neuronal ALS/FTD models and mitigate motor symptoms in an *in vivo* rat ALS model. At high doses, SINE compounds block nuclear egress of an XPO1 cargo reporter, but not at lower concentrations that were associated with neuroprotection. Neither SINE compounds nor leptomycin B, a separate XPO1 inhibitor, enhanced nuclear TDP43 levels, while depletion of XPO1 or other exportins had little effect on TDP43 localization, suggesting that no single exporter is necessary for TDP43 export. Supporting this hypothesis, we find overexpression of XPO1, XPO7 and NXF1 are each sufficient to promote nuclear TDP43 egress. Taken together, our results indicate that redundant pathways regulate TDP43 nuclear export, and that therapeutic prevention of cytoplasmic TDP43 accumulation in ALS/FTD may be enhanced by targeting several overlapping mechanisms.

## Introduction

Amyotrophic lateral sclerosis (ALS) is the most common form of motor neuron disease, affecting approximately 2-3 per 100,000 individuals worldwide^1–3^. Although traditionally described as a pure motor condition that spares cognition^4^, up to half of those diagnosed with ALS exhibit cognitive and behavioral deficits analogous to those found in a separate disorder, frontotemporal dementia (FTD)^5,6^. Supporting a fundamental connection between ALS and FTD, mutations in several genes, including *TARDBP*, *FUS*, *UBQLN2*, *TBK1*, *VCP*, *OPTN*, and *C9orf72*, result in both diseases^7–16^. Moreover, in the majority of those with ALS and FTD, the pathologic hallmark of disease is the cytoplasmic deposition of the nuclear RNA binding protein TDP43^17^, implying a common mechanism underlying these clinically-overlapping neurodegenerative disorders.

The subcellular localization of TDP43 and related RNA binding proteins is critical for the function and the survival of neurons. These proteins contain nuclear localization and nuclear export signals that facilitate rapid trafficking between the nucleus and cytoplasm. Disrupting the TDP43 nuclear localization signal enhances neurotoxicity^18–20^ and, in some cases, mimics the effects of disease-associated mutations in *TARDBP*, the gene encoding TDP43^18^. Strategies that effectively reduce cytoplasmic TDP43 concentrations, including the induction of macroautophagy (one of the major catabolic pathways active within the cytoplasm)^21^, prevent neurodegeneration and extend cellular survival in models of ALS and FTD. These observations underscore the physiologic importance of cytoplasmic protein deposition in the pathogenesis of ALS and FTD.

A growing body of evidence indicates that nucleocytoplasmic transport dysfunction is a conserved pathway underlying neurodegeneration and the age-dependent susceptibility to neurodegenerative diseases^22–27^. Several disease-associated proteins, including fragments of the amyloid precursor protein, huntingtin, and TDP43, misfold into conformations that selectively disrupt nuclear import^28^. Furthermore, reduced expression of nucleocytoplasmic transport components is a defining feature of aging in fibroblasts and neurons directly differentiated from aged fibroblasts^26^. A subtle impairment in nucleocytoplasmic transport with age may predispose neurons to the detrimental effects of pathogenic mutations or proteotoxic stress, potentially explaining the age-dependent onset of neurodegeneration in ALS, FTD, and other neurodegenerative conditions.

TDP43 contains a canonical leucine-rich nuclear export signal (NES)^29,30^ that is predicted to be a substrate of exportin-1 (XPO1/CRM1), a conserved nuclear export factor in mammalian cells^31,32^. We took advantage of novel selective inhibitors of nuclear export (SINE) compounds^33^ to determine whether preventing TDP43 nuclear export might slow or prevent neurodegeneration in models of ALS and FTD. Like the natural XPO1 inhibitor leptomycin B, SINE compounds interact specifically with the cargo binding pocket of XPO1, forming a covalent adduct with XPO1 at cysteine 528^34,35^. This covalent bond is slowly reversible (t_1/2_ of ~24 h), likely contributing to how well the compound class is tolerated in ongoing human and veterinary clinical studies^36^. We therefore conducted a careful study of SINE compounds and their therapeutic potential in both *in vitro* and *in vivo* disease models, testing the ability of these small molecules to prevent cytoplasmic TDP43 mislocalization and extend neuronal survival.

## Results

### Safety of SINE compounds in rodent primary cortical neurons

**Figure 1:**
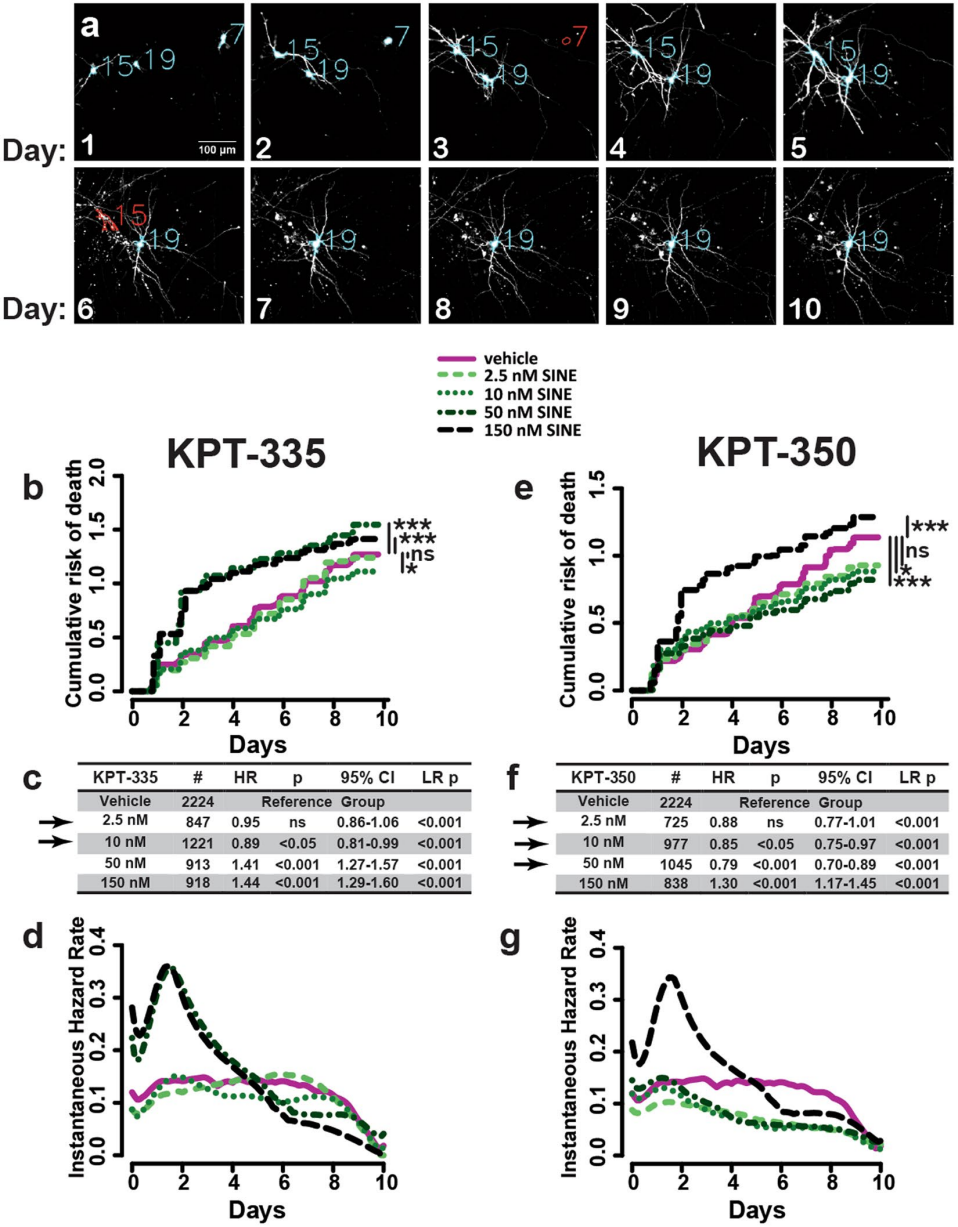
Safety profiling of SINE compounds in rodent primary cortical neurons. (**a**) Primary rodent cortical neurons were transfected with EGFP and imaged every 24 h for 10 days. SINE compounds or vehicle (DMSO) were added after imaging on day 1. Neurons were identified based on morphology (blue outlines) and assigned a unique identifier (blue #). Death (red) is determined by loss of fluorescent intensity (neuron 7, day 3) or changes in morphology (neuron 15, day 6), and time of death for each cell is used to estimate the cumulative (**b**,**e**) and instantaneous (**d**,**g**) risk of death functions. Tables showing neuron number (#), hazard ratio (HR), Cox proportional hazards p value (p), confidence interval (95% CI), and log-rank p value (LR p) are shown in (**c**) and (**f**). Doses considered “safe” are indicated with black arrows. Each condition represents 3-6 combined experiments, minimum 8 technical replicates per experiment.

We first performed dose-finding studies to identify the highest concentration of SINE compounds that can safely be used in cultured mammalian neurons. We tested two different SINE compounds, KPT-335 and KPT-350. Both are selective inhibitors of XPO1, but the former is more potent (IC50 = 33 nM and brain/plasma ratio 1.81; S.T. unpublished observations), while the latter exhibits greater blood brain barrier (BBB) permeability (IC50 = 161 nM and brain/plasma ratio = 2.24)^34^. To screen multiple concentrations of each compound in a rapid and sensitive manner, we took advantage of automated fluorescence microscopy^18,21,37,38^ (Fig. 1a). This technique involves the transient transfection of rodent primary neurons with vectors encoding fluorescent proteins such as enhanced green fluorescent protein (EGFP), enabling their visualization by fluorescence microscopy. The system is fully automated, taking snapshots of thousands of neurons in culture, and is capable of returning to every neuron at any time. The images are analyzed using custom-designed programs that distinguish neurons from debris and glia, assigning each cell a unique identifying number. Cells are tracked longitudinally on a daily basis for 10 days, and cell death is determined prospectively by a set of criteria (rounding of the soma, blebbing, dendritic retraction or loss of fluorescence) that have proven to be sensitive measures of cell death in previous studies^18,39^. Because the event of cell death for the majority of neurons may not be directly witnessed by this method, the time of death is conservatively assigned as the last time the cell was classified as alive.

Using Cox proportional hazards analysis, we estimated the risk of death as a cumulative and instantaneous function for neurons expressing EGFP and treated with vehicle (DMSO) or different concentrations of KPT-335 or KPT-350. Drug was added 24 hours after transfection, after the first round of imaging. Concentrations above 150 nM caused excessive cell death (data not shown), so we focused on 150 nM or less of each compound. For KPT-335 (Fig. 1b–d), significant toxicity was observed at 50 nM and 150 nM. The hazard ratio (HR) listed in Fig. 1c represents a relative risk of death for neurons treated with the indicated dose of SINE compound, relative to control neurons treated with vehicle. By this measure, both 50 nM and 150 nM KPT-335 increased the risk of death by ~40% (Fig. 1b,c), while no toxicity was observed for KPT-335 at 2.5 nM and 10 nM. The instantaneous risk of death, a measure of how likely neurons are to have died at the indicated time^40^ (Fig. 1d), shows a prominent increase in the risk of death for neurons treated with 50 nM and 150 nM KPT-335 that peaks 1 day after application of the compound (corresponding to day 2 of imaging). In contrast, 10 nM KPT-335 slightly but significantly (Fig. 1b,c) reduced the risk of death in comparison to vehicle alone, suggesting a modest protective effect. While 2.5 nM KPT-335 exhibited a small and significant protective effect when measured by the log-rank test (Fig. 1c), we were unable to confirm statistical significance by Cox proportional hazards analysis, which accounts for multiple comparisons among the separate populations^41^.

A similar effect was observed in primary neurons treated with KPT-350 (Fig. 1e–g). Here, however, toxicity was noted only at 150 nM. While 10 and 50 nM KPT-350 extended neuronal survival by 15-20% (Fig. 1e,f), the effect of the 2.5 nM dose was less pronounced and as before, only statistically significant by the log-rank test. The instantaneous risk of death plot (Fig. 1g) demonstrates acute toxicity peaking 1 day after drug application for high doses of KPT-350, analogous to the effect of KPT-335 (Fig. 1d). Protection, or reduced risk of death, was noted for lower doses of KPT-350 beginning approximately 72 h after application of the compound. These results suggest that SINE compounds modestly improve survival in control neurons when used at low doses (≤10 nM KPT-335 and ≤50 nM KPT-350). We therefore selected the lowest 2 doses for investigating the utility of XPO1 inhibition in disease models.

### SINE compounds in models of ALS and FTD

To gauge the therapeutic potential of SINE compounds in ALS and FTD, we employed a neuronal disease model involving overexpression of wild-type (WT) and ALS-associated mutant TDP43^18,21,38^. This model recapitulates key features of ALS and the most common pathologic subtype of FTD (frontotemporal lobar degeneration with TDP43 deposits, or FTLD-TDP), including enhanced neurodegeneration upon expression of disease-associated mutant TDP43. In this system, as in other neurodegenerative diseases characterized by accumulation of an endogenous protein^42,43^, overexpression of WT TDP43 (TDP43^*WT*^) is sufficient to reproduce neuron loss. Similar toxicity arising from TDP43^*WT*^ overexpression has been noted in multiple *in vitro* and *in vivo* model systems^18,44–49^, suggesting a dose-dependent effect of the WT protein that is also seen in other neurodegenerative diseases, including Alzheimer’s and Parkinson’s disease^42,50^. Versions of TDP43 carrying mutations linked to familial ALS and FTD (i.e. A315T) are more toxic at lower doses in comparison to TDP43^*WT*^, and can form insoluble cytoplasmic inclusions^18,45,48,51^.

**Figure 2:**
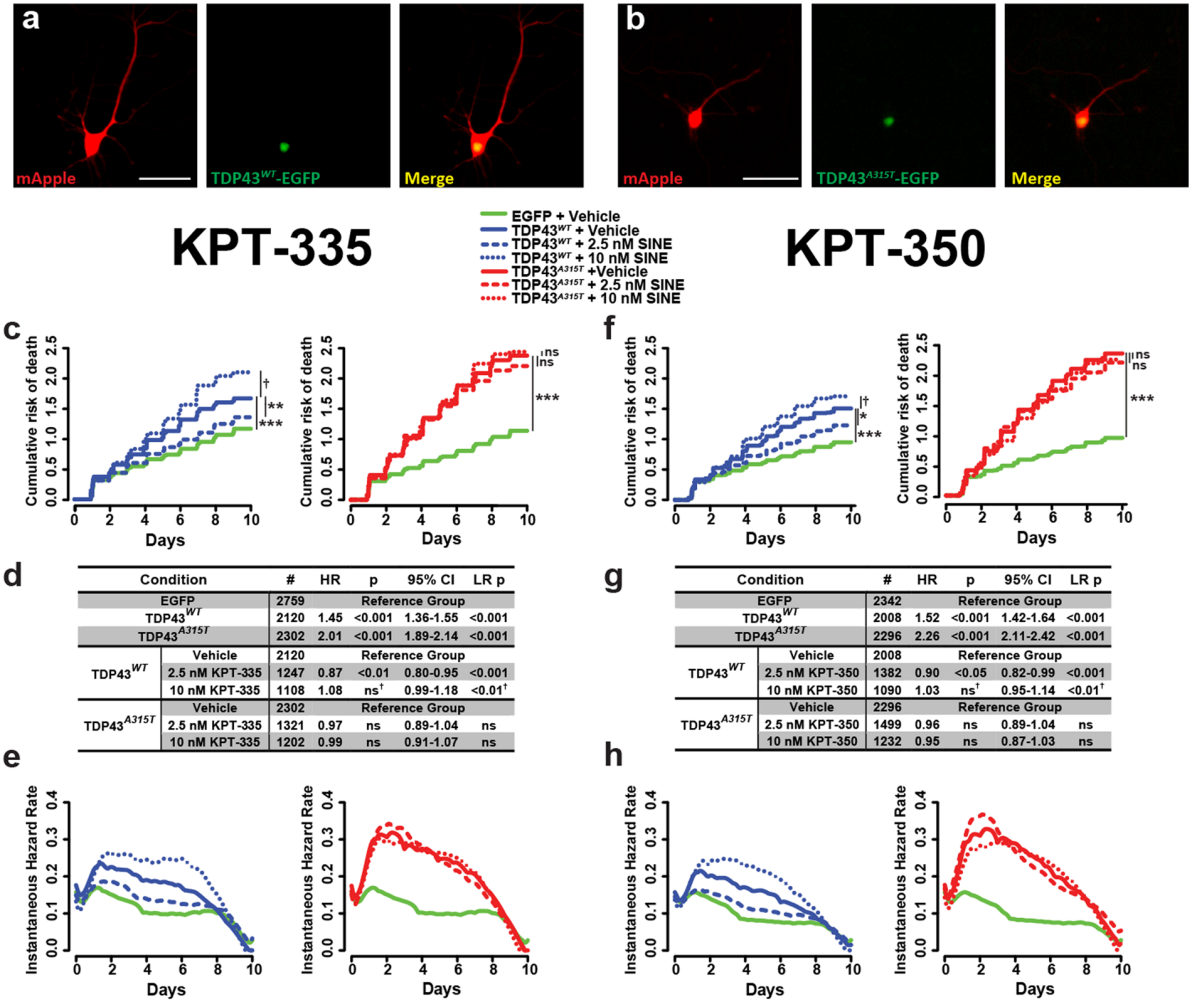
SINE compounds exhibit modest protective effects in models of ALS and FTD. Rodent primary cortical neurons were transfected with TDP43^*WT*^-EGFP (**a**), or the disease-associated mutant TDP43^*A315T*^-EGFP (**b**). Both TDP43^*WT*^-EGFP (solid blue line) and TDP43^*A315T*^-EGFP (solid red line) significantly increased the cumulative (**c**,**f**) and instantaneous (**e**,**h**) risk of death, compared to control neurons expressing EGFP alone (solid green line). Addition of 2.5 nM KPT-335 or KPT-350 (dashed lines) protected against toxicity from TDP43^*WT*^-EGFP (**c**–**h**, blue) but not TDP43^*A315T*^-EGFP (**c**–**h**, red). 10 nM SINE compound (dotted lines) enhanced the risk of death in neurons expressing TDP43^*WT*^-EGFP but not TDP43^*A315T*^-EGFP (LR test **d**,**g**). Neuron number (#), hazard ratio (HR), Cox proportional hazards p value (p), confidence interval (95% CI), and log rank p value (LR p) for each comparison are listed in (**d**) and (**g**). Each condition represents 3 combined experiments, minimum 8 technical replicates per experiment.

For these studies, we transiently transfected primary neurons with vectors encoding the red fluorescent protein mApple, as well as TDP43^*WT*^ or mutant TDP43^*A315T*^ fused to EGFP (Fig. 2a,b). This enabled us to independently track the survival of neurons using mApple as a cellular marker in the red channel, and the expression and subcellular distribution of TDP43-EGFP in the green channel. Neurons were treated with 2.5-10 nM KPT-335 (Fig. 2c–e), KPT-350 (Fig. 2f–h), or vehicle (DMSO) and their survival assessed by automated fluorescence microscopy. As expected, both TDP43^*WT*^-EGFP and TDP43^*A315T*^-EGFP were toxic when overexpressed, but toxicity was more pronounced for TDP43^*A315T*^-EGFP. The lowest dose (2.5 nM) of KPT-335 exhibited slight neuroprotective effects in neurons transfected with TDP43^*WT*^-EGFP, but not in those expressing TDP43^*A315T*^-EGFP (Fig. 2c,d). The reduction in the risk of death with 2.5 nM KPT-335 is evident in both the cumulative (Fig. 2c) and the instantaneous risk of death plots (Fig. 2e). Despite its beneficial effects observed in control neurons expressing EGFP alone (Fig. 1b–d), 10 nM KPT-335 elicited marginal toxicity in TDP43^*WT*^-EGFP expressing neurons that was significant by the log-rank test, but not by Cox proportional hazards analysis (Fig. 2d).

Treating TDP43-expressing neurons with KPT-350 produced similar results (Fig. 2f–h). The 2.5 nM dose of KPT-350 was the most neuroprotective, reducing the risk of death in cells transfected with TDP43^*WT*^-EGFP by ~10% (HR 0.90, p < 0.05 by Cox proportional hazards), while higher doses of KPT-350 trended towards toxicity in these cells. The extension of neuronal survival by KPT-350 was observed by measuring the cumulative (Fig. 2f) and instantaneous risk of death (Fig. 2h) for transfected neurons. As with KPT-335 (Fig. 2c,d), 10 nM KPT-350 demonstrated slight toxicity in TDP43^*WT*^-EGFP expressing neurons that was significant only by the log-rank test, but not Cox proportional hazards analysis (Fig. 2g). In contrast, neither KPT-335 nor KPT-350 significantly affected the survival of neurons expressing TDP43^*A315T*^-EGFP. Together, these data show that low doses (2.5 nM) of KPT-335 and KPT-350 moderately improve neuronal survival in models of ALS and FTD involving the expression of WT but not mutant TDP43.

### Utility of SINE compounds in a neuron model of Huntington’s disease

**Figure 3:**
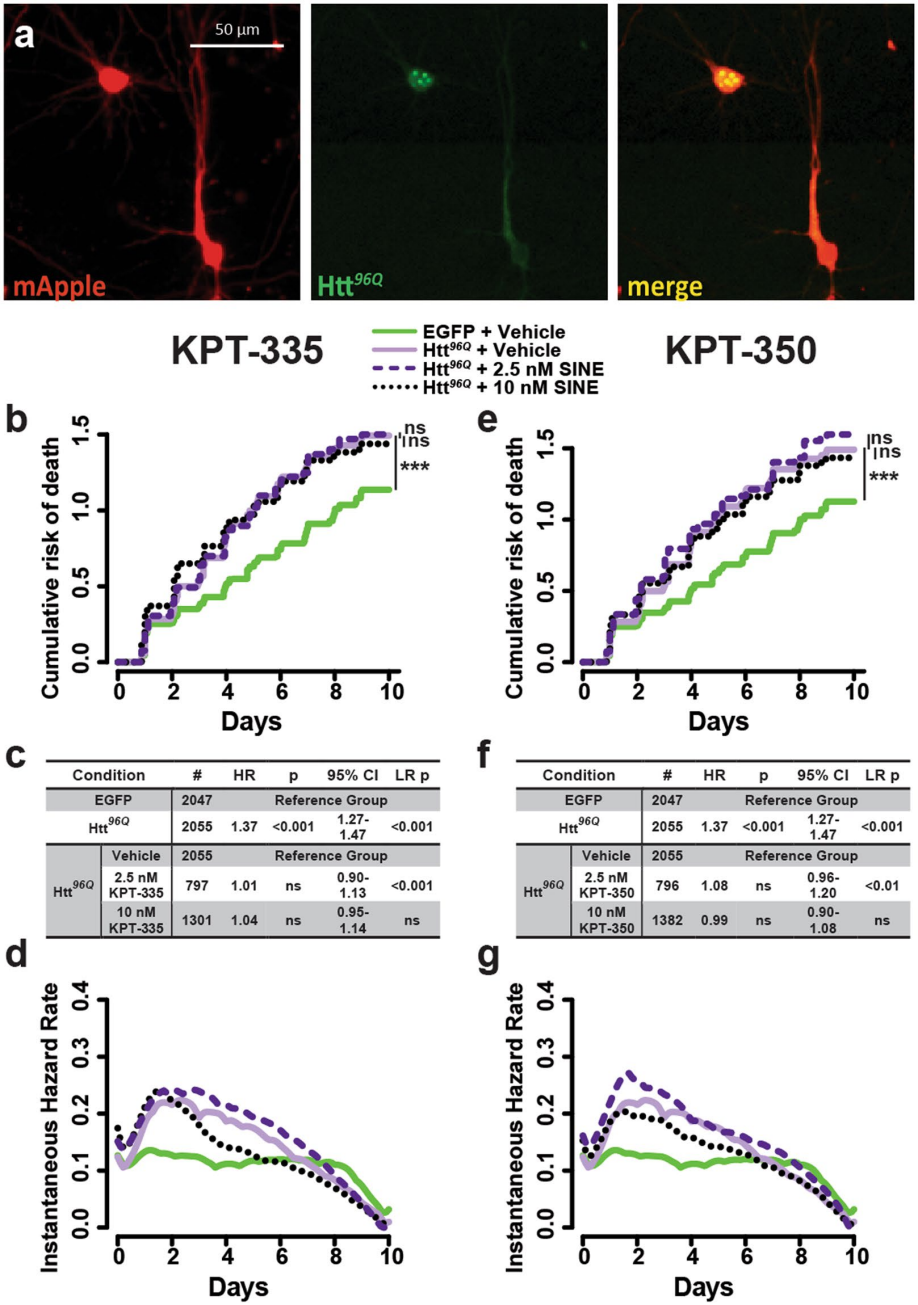
SINE compounds fail to protect against neurodegeneration in a model of Huntington’s disease. (**a**) Rodent primary cortical neurons expressing Htt^*96Q*^-EGFP, a fragment of mutant huntingtin including an expanded 96-residue polyglutamine stretch and fused to EGFP, were imaged by fluorescence microscopy. Following automated survival analysis, cumulative (**b**,**e**) and instantaneous risk of death (**d**,**g**) were determined for Htt^*96Q*^-expressing neurons (purple lines) in comparison to control cells expressing EGFP (green lines). Additional information, including the number of neurons (#), hazard ratio (HR), Cox proportional hazards p value (p), confidence interval (95% CI), and log rank p value (LR p) are shown for all experiments in (**c**,**f**). Each condition represents 3 combined experiments, 8 wells per condition, per experiment.

Given the neuroprotective effects of SINE compounds in control neurons expressing EGFP alone (Fig. 1) and in those transfected with WT TDP43 (Fig. 2), we surmised that SINE compounds might exert a general and non-specific beneficial effect that would extend to neurodegenerative diseases unrelated to ALS or FTD. We therefore turned to a neuronal model of Huntington’s disease, in which neurons overexpress a fragment of mutant huntingtin (Htt) carrying 96 polyglutamine residues fused to EGFP (Htt^*96Q*^-EGFP). In previous studies, neurons expressing this construct exhibit neurodegeneration and the time-dependent formation of nuclear Htt-rich inclusions reminiscent of those found in individuals with Huntington’s disease^37,38,52,53^. Primary rodent cortical neurons were transfected with vectors encoding the cellular marker mApple, and either Htt^*96Q*^-EGFP or EGFP alone, treated with KPT-335 or -350, and imaged by automated microscopy (Fig. 3a). As expected, Htt^*96Q*^-EGFP expression increased the risk of death by ~40% in comparison to EGFP alone (Fig. 3b–g). Neither KPT-335 nor KPT-350 effectively promoted neuronal survival in this model, suggesting that their effects are likely not generalizable to all neurodegenerative diseases. However, as toxicity in this model is more likely to correlate with increased, rather than decreased nuclear retention of mutant Htt^54^, it is possible that the modest neuroprotection afforded by SINE compounds may be offset by enhanced toxicity of nuclear mutant Htt.

In contrast to our observations, a prior study^55^ noted that KPT-350 effectively prevented neurodegeneration in a Huntington’s disease model. To determine the potential reasons for such discrepancies, we sought to replicate the conditions in which the previous investigations were performed. The most notable differences involved the timing of drug application and the method for determining cell death: while Grima *et al*.^55^ added 10–100 nM KPT-350 at the time of transfection and quantified fluorescently labeled cells exhibiting pkynosis to estimate cell death, we applied SINE compounds 24 h following transfection and assessed neuronal survival by automated microscopy. When we mimicked the prior study by applying SINE compounds to neurons *at the time of transfection*, then monitored neuronal survival by automated microscopy, we noted a dose-dependent decrease in the number of cells detected 24 h post transfection (Fig. S1a–c), likely reflecting the peak of cell death observed previously (Fig. 1) — in primary neurons, SINE compound-induced toxicity is rapid, peaking within 24 h of application and resolving within 48 h. A similar effect on the number of neurons detected 24 h after transfection was observed for both KPT-335 and KPT-350, and was equally prominent in neurons expressing EGFP and EGFP-tagged versions of TDP43^*WT*^, TDP43^*A315T*^ (Fig. S1a–c) and Htt^*96Q*^ (Fig. S1d,e). We suspected that this early drug-dependent toxicity might skew the results of survival analysis if not considered when assessing the effect of SINE compounds on neurodegeneration. To test this possibility, we tracked neuronal survival by automated microscopy beginning 24 h or 48 h following application of SINE compounds, which were added at the time of transfection. In doing so, we recapitulated the apparent neuroprotective effect of SINE compounds in cells overexpressing Htt^96Q^-EGFP (Fig. S1f–h). The apparent neuroprotective effect was particularly evident when the analysis began 48 h following transfection and drug addition, after the peak of SINE compound-induced toxicity had passed. Taken together, these data confirm the initial toxicity of SINE compounds when applied to neurons, an effect that may misrepresent their neuroprotective potential unless properly taken into account.

### Evaluation of SINE compound efficacy in an *in vivo* ALS/FTD model

**Figure 4:**
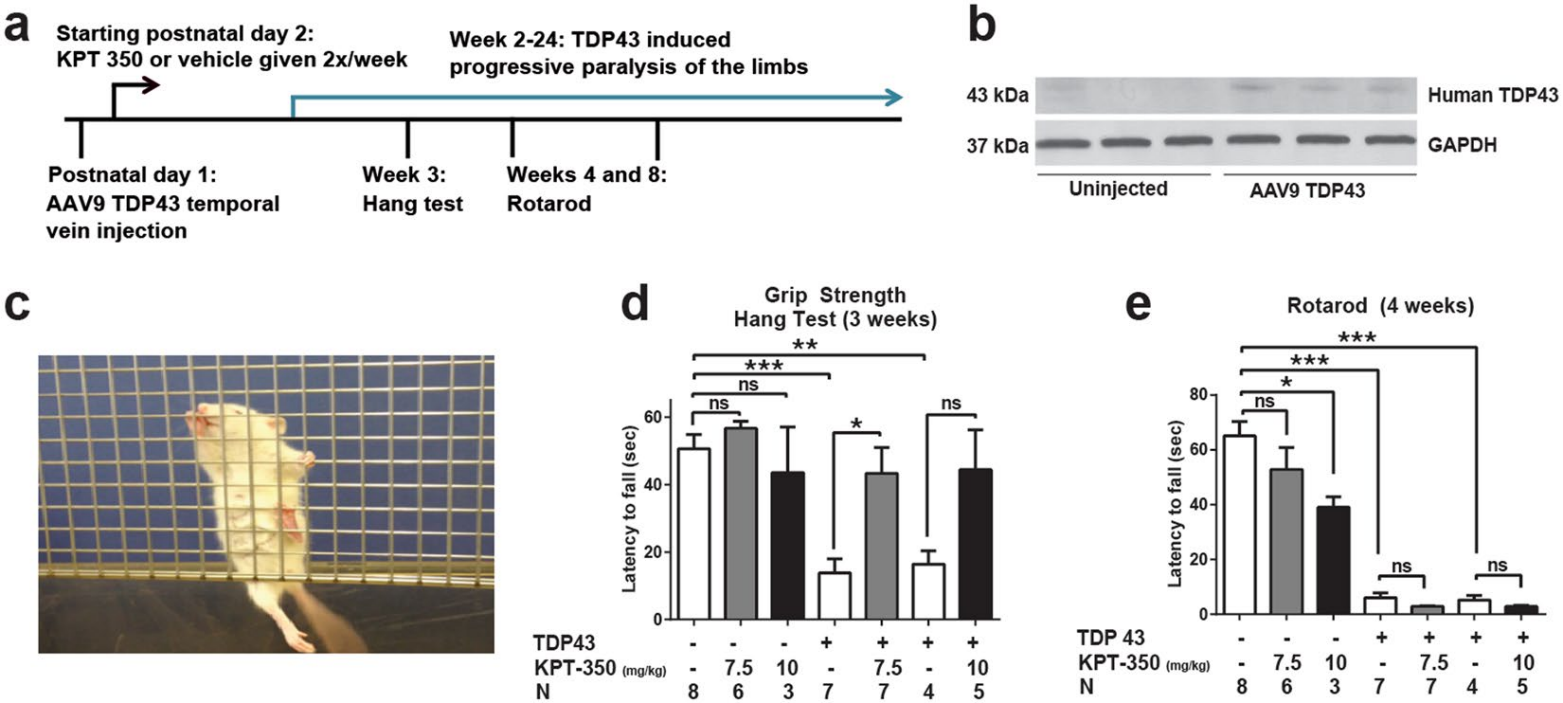
SINE compounds partially rescue motor deficits in an animal model of ALS/FTD. (**a**) Schematic timeline for *in vivo* experiments. (**b**) Western blot demonstrating the expression of human TDP43 within the brains of AAV9-injected animals, which we have previously shown is largely restricted to neuronal cell types (Jackson *et al*.^59^). Full-length blots appear in Fig. S1. (**c**) The hang test measures the time it takes for animals to fall from grid-like scaffold when it is lifted and tilted. A single representative animal administered AAV9-TDP43 is shown; note the inability of the hind limbs to clasp the wire mesh. (**d**) TDP43 expression impaired motor performance on the hang test at 3 weeks, an effect that could be mitigated by 7.5 mg kg^-1^ KPT-350. (**e**) Rotarod testing at 4 weeks of age showed prominent deficits associated with TDP43 expression that were resistant to treatment with SINE compounds. n, number of animals per condition. ns, not significant, mean ± s.e.m. *p < 0.05, **p < 0.01, ***p < 0.001, one-way ANOVA with Bonferroni correction.

We also explored whether XPO1 inhibition could prevent neurodegeneration arising from TDP43 accumulation in animals. For this purpose, we used a rat model of TDP43-induced paralysis^56–59^. Human WT TDP43 cDNA was delivered to neonatal rats via an intravenous injection of recombinant adeno-associated virus serotype 9 (AAV9), yielding consistent limb paralysis that gradually progresses over the ensuing 2-16 weeks^57,59^. To determine if SINE compounds prevented clinical manifestations of TDP43-related toxicity, transduced rats were treated at day 2 with one of two separate doses of KPT-350 (7.5 mg kg^−1^ and 10 mg kg^−1^) (Fig. 4a), as this compound exhibits greater blood brain barrier (BBB) permeability, and identical doses of KPT-350 proved beneficial in prior studies involving other neurological disorders^34,60^. After verifying TDP43 expression within the central nervous system of transduced rats (Fig. 4b and Fig. S2), we measured motor function of treated and untreated animals using the hang test (Fig. 4c) and the rotarod at 3–4 weeks of age. As expected, TDP43-transduced rats exhibited a dramatically shortened latency to fall on both tests, in comparison to uninjected controls. Treatment with 7.5 mg kg^−1^ KPT-350 almost completely rescued the deficits in grip strength as measured by the hang test at 3 weeks (Fig. 4d). Although there was a trend towards improvement on the hang test with the 10 mg kg^−1^ dose, the effect was non-significant. In addition, neither the 7.5 mg kg^−1^ nor the 10 mg kg^−1^ dose affected rotarod performance at 4 weeks (Fig. 4e) or 8 weeks (Fig. S3a). We also observed dose-dependent weight loss in KPT-350-treated animals (Fig. S3b), consistent with prior reports of anorexia and weight loss following treatment with SINE compounds^36^. Thus, SINE compounds partially rescue motor function in TDP43-overexpressing animals, although their use is limited by weight loss.

### Subcellular protein localization in response to XPO1 inhibition

**Figure 5:**
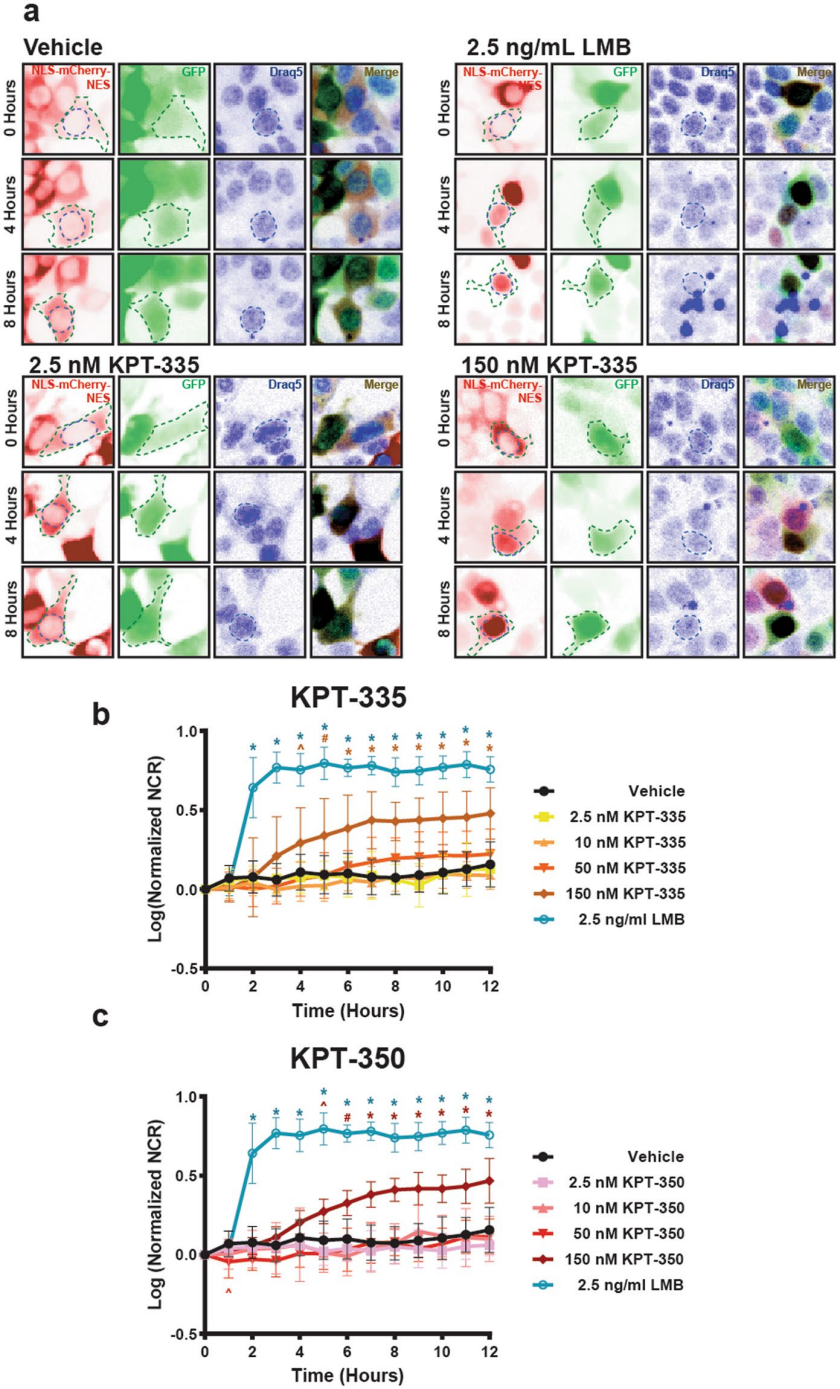
Nuclear localization of a reporter construct is enhanced by XPO1 inhibition. (**a**) The NLS-mCherry-NES fusion protein (inverted images false colored red, left panels) is localized primarily within the cytoplasm of HEK293 cells at steady-state, reflecting the dominance of the NES. Cells were cotransfected with EGFP as a cellular marker (green), and treated with the cell permeable infrared nuclear dye Draq5 (blue). Addition of 2.5 ng ml^−1^ leptomycin B (LMB) results in nuclear localization of the reporter by 4 h. At the 150 nM dose, KPT-355 and KPT-350 also enhanced nuclear reporter localization, albeit more slowly and less effectively than LMB. Low doses of KPT-335 and KPT-350 evoked no measurable changes in NLS-mCherry-NES localization. (a–**c**) The nuclear/cytoplasmic ratio (NCR) for the reporter was calculated as a function of time for HEK293 cells treated with LMB, SINE compounds or vehicle, and log transformed prior to plotting. Representative graphs in (**b**,**c**) constructed from n = 10 cells per condition, mean ± s.d., *p < 0.001, ^#^p < 0.01, ^p < 0.05, one-way ANOVA with Dunnett’s correction.

Inhibition of XPO1 by SINE compounds is expected to enhance nuclear localization for proteins that rely upon XPO1 for nuclear export. To verify the activity of these compounds in living cells, we applied SINE compounds to HEK293 cells expressing a red fluorescent protein (mCherry) carrying a strong leucine-rich nuclear export signal recognized by XPO1, as well as a weak nuclear localization signal (NLS-mCherry-NES)^61^. Cells were cotransfected with EGFP to mark the cytoplasm and stained with the cell permeable nuclear marker Draq5. We then imaged the cells by fluorescence microscopy at regular intervals following the addition of SINE compounds (Fig. 5a). As a positive control, we used leptomycin B (LMB), a potent and irreversible XPO1 inhibitor^32,62^. Upon addition of 2.5 ng/ml LMB, the NLS-mCherry-NES reporter rapidly accumulated within the nucleus of transfected HEK293 cells; by 4 h, nearly all of the protein was found within the nucleus (Fig. 5a–c). Low-dose (2.5 nM) KPT-335 and KPT-350 produced no discernable change in reporter localization up to 12 h after drug addition, and 10 and 50 nM of the SINE compounds were equally ineffective. Only 150 nM of KPT-335 and KPT-350 elicited measurable increases in reporter nuclear localization (Fig. 5a–c). This effect was first evident 4 h after KPT-335 addition, reaching a peak by 7 h. Consistent with its enhanced XPO1 binding affinity, KPT-335 elicited more consistent and pronounced nuclear localization of the reporter than did KPT-350. These results suggest that SINE compounds are incapable of modulating nucleocytoplasmic transport at the low doses that were associated with neuroprotection in previous experiments (Figs 1, 2).

**Figure 6:**
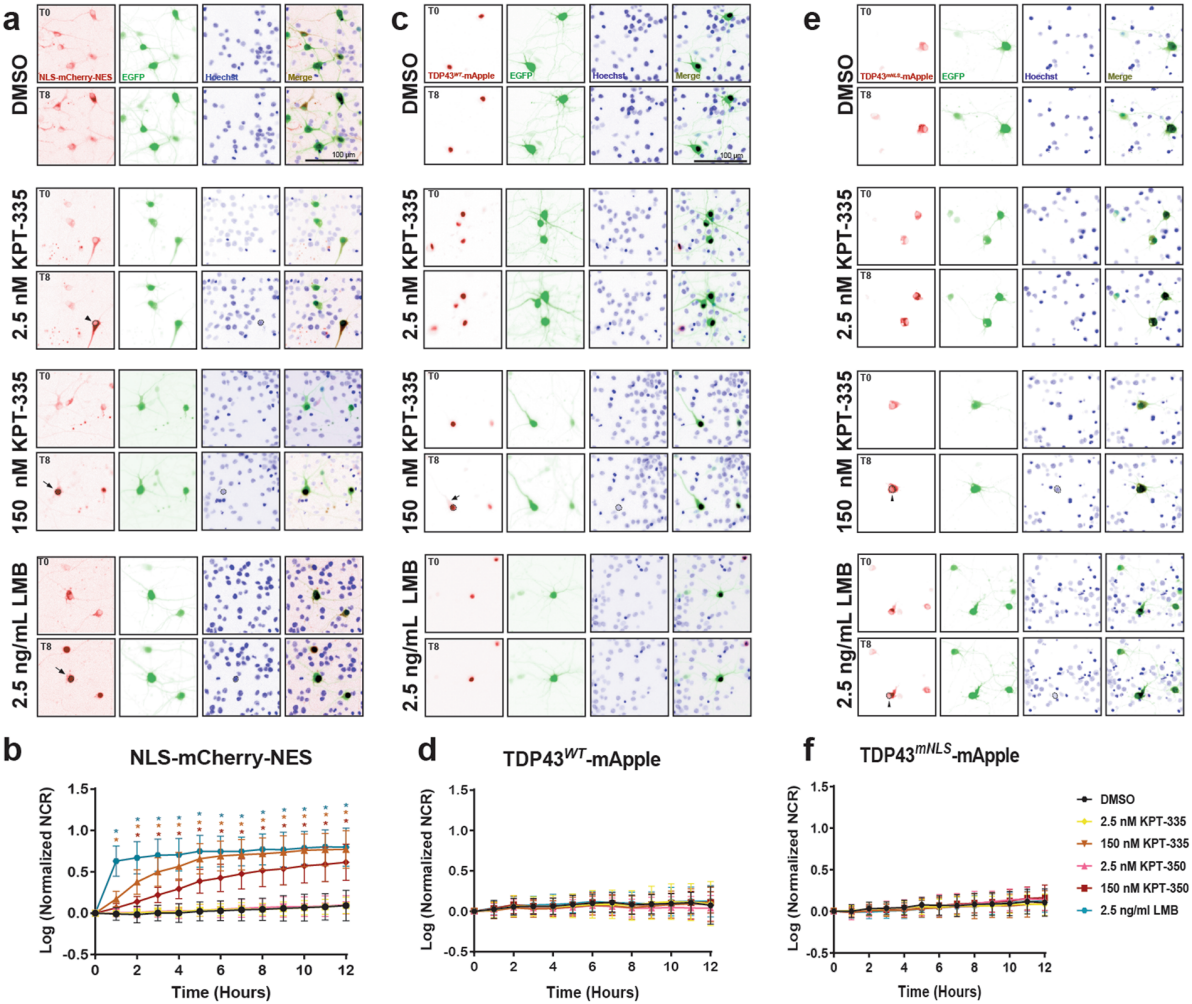
XPO1 inhibition has no detectable effect on the subcellular distribution of TDP43. (**a**) Rodent primary cortical neurons were transfected with EGFP and NLS-mCherry-NES, imaged at baseline, treated with vehicle, LMB or SINE compounds, and imaged at 1 h intervals for 12 h. Representative images (false colored and inverted for clarity) are shown before (0) and 8 h after drug addition. Nuclear retention of the reporter construct is observed with LMB treatment or high (150 nM) doses of SINE compounds (black arrows, left panel at T8) but not at low (2.5 nM) doses (black arrowhead, left panel at T8). (**b**) LMB significantly increases the reporter nuclear to cytoplasmic ratio (NCR) by 1 h. At 150 nM, both SINE compounds significantly increase nuclear localization of NLS-mCherry-NES, with slightly slower kinetics than LMB. No change in reporter localization was observed with 2.5 nM of either SINE compound. (**c**,**d**) Following transfection with EGFP and TDP43^*WT*^-mApple, primary neurons were treated with the indicated compound and imaged by longitudinal microscopy. TDP43^*WT*^-mApple is predominantly nuclear at steady state, and SINE compounds have no observable effects on its localization. Representative image in (**c**) shows that no decrease in cytoplasmic TDP-43^*WT*^ –mApple levels (black arrow, left panel at T8) can detected in cells treated with 150 nM KPT-335 8 h after treatment. (**e**,**f**) In primary cortical neurons, TDP43^*mNLS*^-mApple is predominantly cytoplasmic at steady-state. XPO1 inhibition fails to significantly increase TDP43^*mNLS*^-mApple NCRs at any tested dose. Nuclear clearance of TDP43^*mNLS*^-mApple was notable in treated cells, even 8 h after drug addition (black arrowheads, left panels at T8). Representative plots in **b**, **d**, **f** were constructed from n = 43-45 cells per condition (13-15 cells per condition, 3 replicates each), mean ± s.d., *p < 0.001, ^#^p < 0.01, ^p < 0.05, one-way ANOVA with Dunnett’s correction.

To determine if these observations extended to mature post-mitotic neurons, we transfected rodent primary cortical neurons with vectors encoding EGFP and NLS-mCherry-NES, applied LMB, SINE compounds or vehicle, and imaged the cells by automated microscopy (Fig. 6a,b). As with HEK293 cells, treating transfected neurons with LMB produced a rapid relocalization of NLS-mCherry-NES from the cytoplasm to the nucleus. At low doses (2.5 nM), neither KPT-335 nor KPT-350 provoked a significant change in the subcellular distribution of the reporter. Higher doses of the SINE compounds (150 nM) resulted in a subtle and gradual increase in nuclear reporter localization, with peak effects observed 8 h or more after drug addition. Once more, KPT-335 caused a more conspicuous change in reporter localization than did KPT-350, indicative of more potent XPO1 inhibition by KPT-335. These results are consistent with those from HEK293 cells, suggesting that high-dose but not low-dose SINE compounds effectively enhance nuclear localization of XPO1 substrates. Our observations also show that XPO1-dependent nucleocytoplasmic transport machinery is conserved between primary rodent neurons and HEK293 cells.

TDP43 carries a leucine-rich sequence of the type recognized by XPO1^29,30^. The residues comprising the putative signal sequence (amino acids 239-243 and 248-250) are surface exposed (Fig. S4), suggesting that they may serve as a valid recognition signal for XPO1. However, no investigations to date have examined the functional localization of TDP43 in response to XPO1 inhibition. We sought to determine if TDP43 undergoes XPO1-mediated nucleocytoplasmic transport through two complementary approaches. First, we treated rodent primary cortical neurons with vehicle (DMSO) or the safest dose of SINE compounds used in disease models (Fig. 2), and analyzed the amount of endogenous TDP43 within the nuclear and cytoplasmic fractions of treated neurons by immunoblotting (Fig. S5a–c). Low-dose KPT-335 and KPT-350 had no observable effect on the subcellular distribution of endogenous TDP43 or Hsc70, a documented XPO1 substrate^63,64^. Higher concentrations of SINE compounds also failed to enhance nuclear TDP43 localization, but were limited by dose-dependent cytotoxicity (data not shown). Similarly, no change in the localization of TDP43 was noted in the brains of rats transduced with AAV-TDP43 and treated with KPT-350, in comparison to those treated with vehicle alone (Fig. S5d–e).

In parallel, we also measured the effect of SINE compounds on TDP43 localization in living neurons by fluorescence microscopy. Primary rodent cortical neurons were transfected with vectors encoding EGFP and either NLS-mCherry-NES (Fig. 6a,b) or TDP43^*WT*^-mApple (Fig. 6c,d), treated with LMB, SINE compounds or vehicle, and imaged by automated microscopy at regular intervals over a 12 h period. Despite their ability to prevent nuclear export of the NLS-mCherry-NES reporter (Fig. 6a,b), neither LMB nor the SINE compounds significantly affected TDP43^*WT*^-mApple distribution in neurons (Fig. 6c,d). This observation suggests that TDP43 may not be an XPO1 cargo protein, or that we were unable to accurately detect changes in TDP43^*WT*^-mApple localization with XPO1 inhibition because TDP43 is predominantly nuclear at steady state. To distinguish among these possibilities, in place of TDP43^*WT*^-mApple we used TDP43^*mNLS*^-mApple, which exhibits impaired but not abolished nuclear import. This construct contains point mutations disrupting the first half of the bipartite nuclear localization signal (NLS) (82KRK85 > 82***GGG***85), resulting in a predominantly cytoplasmic distribution^18,29^, much like NLS-mCherry-NES (Fig. 6e). However, we noted no significant increase in TDP43^*mNLS*^-mApple nuclear localization upon application of LMB and the SINE compounds to transfected neurons, even at higher doses (Fig. 6f). Together, these data imply that TDP43 localization is unaffected by XPO1 inhibition.

### Subcellular protein localization in response to XPO1 depletion or overexpression

We also considered the possibility that TDP43 may utilize multiple pathways for nuclear export. To address this hypothesis, we designed a system to quickly and efficiently monitor TDP43 distribution in a large number of cells. We modified our automated survival analysis platform to *i*) identify both the nuclear compartment (based on the signal from a cell-permeable nuclear marker) and the cytoplasm (as identified by a diffuse fluorescent protein) for each cell (Fig. S6b–e); *ii*) exclude dead or dying cells based on nuclear pyknosis; *iii*) calculate nuclear and cytoplasmic TDP43 intensities for individual neurons; and *iv*) report their ratio (Fig. S6b–e,h,i). In comparison with manual analysis (Fig. S6a,f,g), the automated system consistently selects a more conservative cytoplasmic outline but is equally accurate in identifying changes in TDP43 distribution in response to different experimental conditions (compare Fig. S6f and h vs. g and i). These promising results confirm the utility of the automated system for assessing the distribution of TDP43 and other proteins in living neurons.

**Figure 7:**
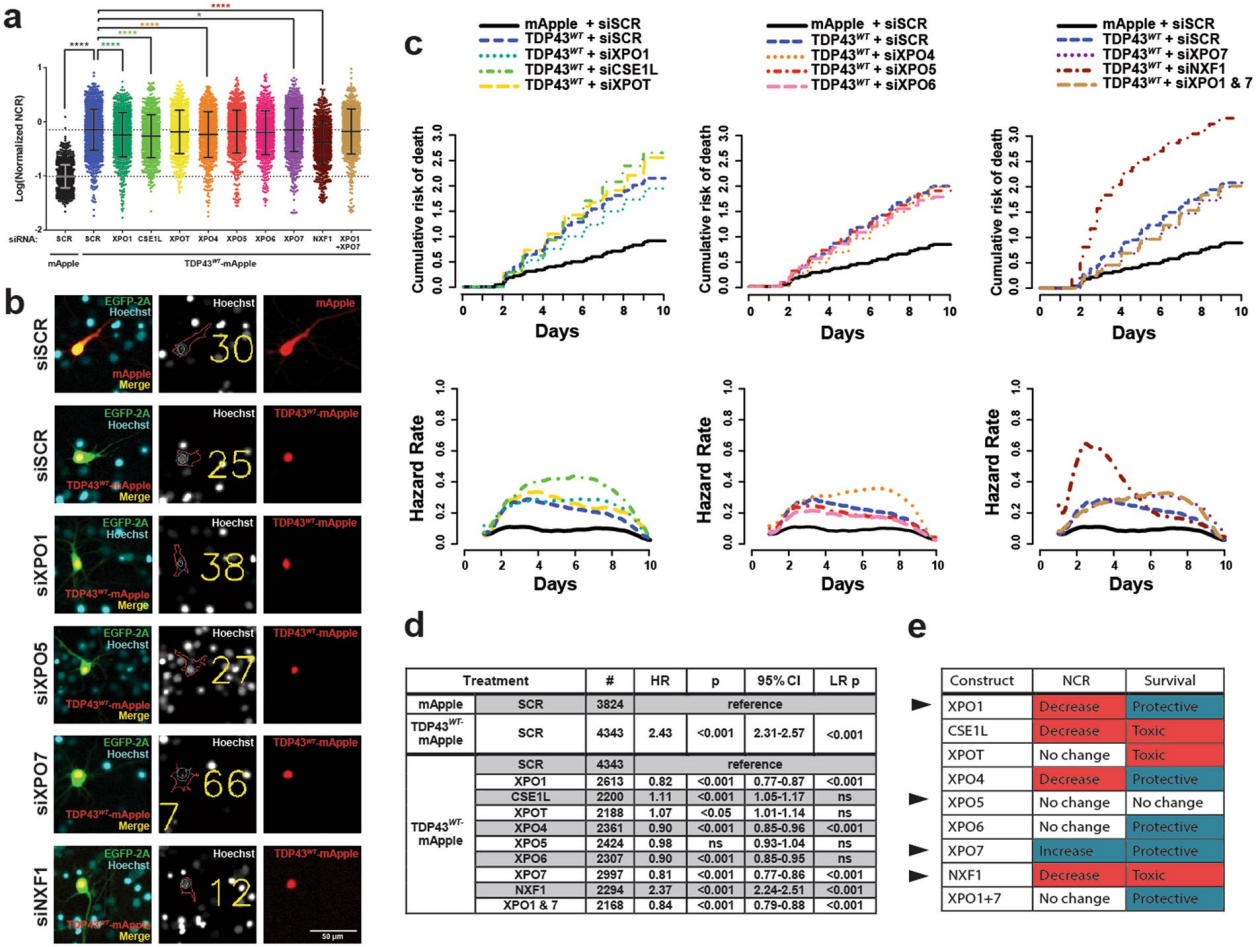
siRNA depletion of export proteins has limited effects on TDP43 localization. (**a**) The distribution of TDP43^*WT*^-mApple, as estimated by the nuclear to cytoplasmic ratio (NCR) was measured 48 h after transient transfection with siRNA targeting nuclear exporters in primary rat cortical neurons. Statistical significance was calculated using 2-way ANOVA with Tukey’s adjustment. Error bars in (**a**) show mean ± s.d. (**b**) Automated analysis of subcellular protein distribution. Center panels demonstrate areas selected to measure average nuclear (within green ellipse) and cytoplasmic (between blue and red bounding lines) intensity. The effect of siRNA depletion on neuronal survival is plotted in (**c**). Depletion of XPO1, XPO7, or both XPO1 & 7 reduce the risk of death in transfected neurons by 20%, while NXF1 depletion more than doubles the risk of death. Cumulative hazard shown on the left, and instantaneous hazard on the right. Additional information, including the number of neurons (#), hazard ratio (HR), Cox proportional hazards p value (p), confidence interval (95% CI), and log rank p value (LR p) are shown for all experiments in (**d**). N = cells per condition (8-16 technical replicates per condition per experiment, minimum 3 experiments per condition). NCR was normalized to control for each biological replicate before pooling. (**e**) Summary of localization and survival effects. Black arrows indicate exporters chosen for further study.

We took advantage of this platform to investigate alternative mechanisms of TDP43 nuclear export. We first asked if any single exporter was necessary for TDP43 nuclear egress. For these experiments, we systematically knocked down all 7 nuclear exporters and NXF1, a nuclear export factor unrelated to the karyopherins that has previously been linked to TDP43 transport^25^. We assessed TDP43^*WT*^-mApple localization in rodent primary neurons 48 hours after treatment with pooled siRNAs targeting each exporter using our automated system for measurement of subcellular protein distribution. Verification of knockdown efficiencies revealed a 37-59% reduction in average exporter transcript abundance (Fig. S7). We noted small but significant reductions in the mean TDP43^*WT*^-mApple nucleocytoplasmic ratio (NCR) upon knockdown of XPO1, CSE1L, XPO4, and NXF1 (Fig. 7a). Knockdown of XPO7 was the sole manipulation that significantly increased the mean TDP43^*WT*^-mApple NCR in transfected cells (Fig. 7a,b); even so, the magnitude of the change (1%) suggested that redundant mechanisms participate in TDP43 nuclear egress.

Given previous evidence indicating a proportional relationship between cytoplasmic TDP43 localization and neurodegeneration^18^, we also tracked the survival of primary neurons transfected with TDP43^*WT*^-mApple and siRNA targeting individual nuclear exporters (Fig. 7a–d). Consistent with the effect of XPO7 knockdown on TDP43^*WT*^-mApple distribution, siRNA targeting XPO7 reduced the risk of death in TDP43-expressing neurons by approximately 20% (Fig. 7d). We observed a similar amelioration of TDP43-mediated toxicity upon XPO1 knockdown, despite the small decrease in TDP43^*WT*^-mApple NCR in these cells. This modest neuroprotection mirrors that afforded by SINE compounds (Fig. 2), suggesting that genetic and pharmacologic inhibition of XPO1 are both capable of mitigating TDP43-related cell death independent of their effects on TDP43 localization. The combined knockdown of XPO1 and XPO7 showed no additive effect (HR of 0.84 compared to 0.82 and 0.81, Fig. 7d), indicating an upper threshold of neuroprotection afforded by this mechanism. XPO4 depletion was also modestly protective, reducing the risk of death by 10%. In contrast, NXF1 depletion produced a striking exacerbation of TDP43-dependent toxicity, more than doubling the cumulative risk of death.

To further explore the possibility that TDP43 undergoes nuclear egress by overlapping pathways, we overexpressed a subset of exporters with experimental or predicted effects on TDP43 nucleocytoplasmic transport, and assessed the subcellular distribution of TDP43^*WT*^-mApple in transfected primary neurons. For these experiments, we selected 4 distinct exporters for overexpression: XPO7, because knockdown of this exporter increased TDP43^*WT*^-mApple NCR and reduced TDP43-mediated neurodegeneration (Fig. 7); XPO1, due to the putative XPO1 recognition motif in TDP43 (Fig. S4), as well as the subtle neuroprotective effects of SINE compounds (Fig. 1) and XPO1 depletion (Fig. 7) in TDP43-overexpressing neurons; NXF1, which dramatically enhanced TDP43-related toxicity and reduced the TDP43^*WT*^-mApple NCR in transfected neurons (Fig. 7); and XPO5 as a control, since knockdown of this protein failed to affect toxicity or localization of TDP43^*WT*^-mApple (Fig. 7). To minimize the potential for steric hindrance from fluorescent tags, we chose to use untagged exporter proteins in our overexpression assays. XPO1, XPO5, and NXF1 were each appended to the EGFP open reading frame with a self-cleaving viral 2A sequence between the exporter and the fluorophore, enabling equimolar expression of separate polypeptides from the same mRNA transcript^65,66^. Untagged XPO7 was transfected concurrently with a plasmid containing the EGFP cell marker, and its simultaneous expression confirmed by immunocytochemistry (Fig. S8a,b).

**Figure 8:**
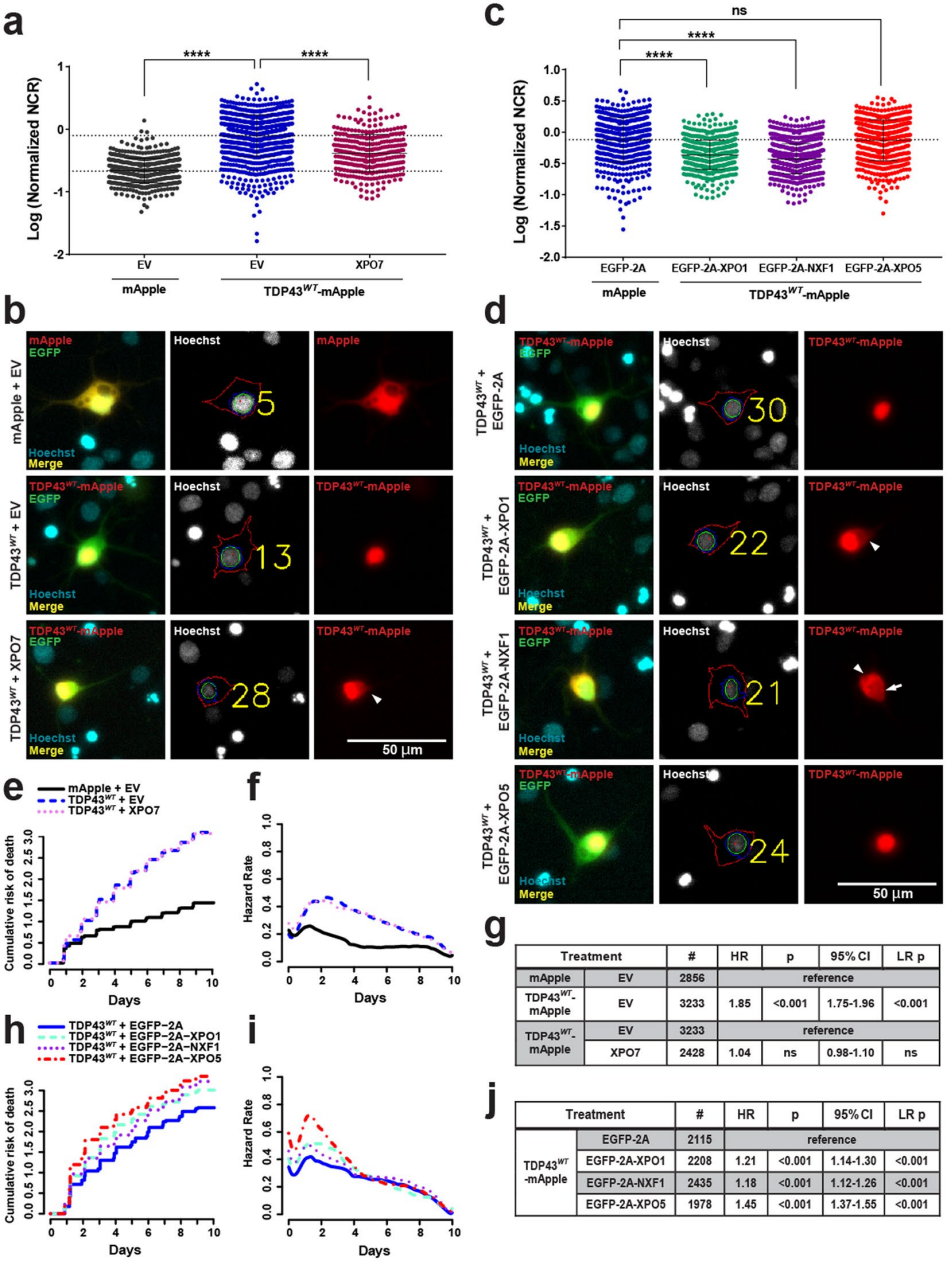
XPO1, XPO7 or NXF1 overexpression enhances cytoplasmic TDP43 localization. (**a**,**b**) Overexpression of untagged XPO7 in rodent primary neurons effectively reduces the nuclear to cytoplasmic ratio (NCR) of TDP43^*WT*^-EGFP compared to cells transfected with empty vector (EV). (**c**,**d**) Overexpression of either XPO1 or NXF1 appended to the self-cleaving EGFP-2A peptide also results in significant reductions in TDP43^*WT*^-mApple NCR, while XPO5 overexpression has no effect on TDP43^*WT*^-mApple localization. Arrowheads indicate cytoplasmic TDP43 accumulation, while arrows highlight nuclear clearance of TDP43. (**e–g**) XPO7 overexpression has little effect on TDP43-dependent toxicity. Cumulative and instantaneous hazards shown in (**e**) and (**f**), respectively, and additional data, number of neurons (#), hazard ratio (HR), Cox proportional hazards p value (p), confidence interval (95% CI), and log rank p value (LR p) are indicated in (**g**). XPO1, XPO5 and NXF1 potentiate the toxicity of TDP43^*WT*^-mApple overexpression (**h**–**j**). Data in **a-j** represent 3 biological replicates per condition, minimum 8 wells per replicate/condition. The NCR was normalized to control for each biological replicate before pooling, and plotted as mean normalized log (NCR) ± s.d. ****p < 0.0001, 2-way ANOVA with Tukey’s adjustment.

Consistent with the hypothesis that TDP43 is transported out of the nucleus via multiple pathways, overexpression of XPO7, XPO1 and NXF1, but not XPO5, significantly decreased the mean TDP43^*WT*^-mApple NCR in transfected primary neurons (Fig. 8a,c). Cytoplasmic TDP43^*WT*^-mApple was clearly visible in cells expressing XPO7, XPO1 and NXF1 (Fig. 8b,d), but nuclear TDP43^*WT*^-mApple exclusion was noted only in NXF1-transfected cells. In contrast, XPO5 had no measurable effect on TDP43 localization. As expected based on the established relationship between cytoplasmic TDP43 redistribution and neurodegeneration^18^, XPO1 and NXF1 exacerbated TDP43-dependent toxicity in transfected neurons (Fig. 8e–j). However, XPO5 overexpression also increased the cumulative and instantaneous risk of death without affecting TDP43^*WT*^-mApple localization, suggesting TDP43-independent toxicity. Supporting this notion, exporter overexpression was significantly toxic in the absence of TDP43 (Fig. S8). Although overexpression of XPO7 alone increased the cumulative risk of death by nearly 100%, it had little effect on TDP43-mediated toxicity in primary neurons (Fig. 8g). Taken together, these data suggest that, while TDP43 is indeed a cargo of XPO1, it is also exported from the nucleus via interactions with multiple transporters, including XPO7 and NXF1.

## Discussion

Here, we conducted a careful study of selective XPO1 inhibition and nuclear TDP43 export in ALS and FTD disease models. Our observations indicate that, within a defined therapeutic window, SINE compounds modestly promote neuronal survival in primary neurons, and partially rescue a motor phenotype in rats overexpressing the RNA binding protein TDP43. However, the therapeutic index for XPO1 inhibition via SINE compounds was narrow, and significant neurotoxicity was observed for the doses that effectively blocked nuclear export. TDP43 localization was largely unaffected by SINE compounds, particularly at the low doses that were safe in neurons. Our data suggest that TDP43, like other essential RNA binding proteins^67^, is trafficked through partially redundant and overlapping pathways, ensuring that TDP43 nuclear export will proceed unchecked even if one or more pathways are blocked. Furthermore, the modest beneficial effects of SINE compounds in ALS/FTD models are likely to be independent of TDP43 localization or nuclear export itself.

Although we observed no effect of SINE compounds on TDP43 distribution, low doses of these compounds moderately extended neuronal survival in primary neurons overexpressing TDP43^*WT*^-EGFP, and KPT-350 partially rescued motor phenotypes in an animal model of ALS involving TDP43 overexpression throughout the CNS. Because cytoplasmic TDP43 deposition is characteristic of ~95% of all ALS and >50% of FTD^14,17,68,69^, in most cases reflecting dysregulation of the *wild-type* protein, these results may still have relevance for both ALS and FTD. The *in vivo* doses used here (7.5–10 mg kg^−1^) fall within the range reported by recent investigations that demonstrated neuroprotective properties of SINE compounds in animal models of inflammatory demyelination and traumatic brain injury^34,60^. These prior studies hinted at the possibility that XPO1 inhibition might be generally neuroprotective. Our results, however, suggest that KPT-350 is incapable of fully preventing neurodegeneration in TDP43-overexpressing rats. Moreover, SINE compounds elicited substantial neurotoxicity at doses associated with impaired nuclear export of a reporter protein, failed to prevent neurodegeneration in response to the ALS-associated TDP43^*A315T*^-EGFP, and were ineffective at promoting neuronal survival in a Huntington’s disease model.

In a related study^55^, KPT-350 displayed neuroprotective effects in a primary neuron Huntington’s disease model. Several differences account for this discrepancy. First, we added SINE compounds 24 h following transfection to mimic treatment of the disease rather than prevention, while Grima *et al*.^55^ applied KPT-350 immediately following transfection with Htt^*96Q*^-EGFP. We show that applying SINE compounds at the time of transfection elicits substantial early toxicity that, if not recognized, may inappropriately skew the results of subsequent survival analyses. Second, we directly assessed the survival of transfected neurons by visualizing cellular morphology, rather than relying on nuclear condensation of fixed cells as a proxy. Lastly, we followed neurons periodically for 10 days instead of 2, minimizing the significance of transient effects and enabling us to capitalize on the increased statistical power of longitudinal survival analysis.

XPO1 is responsible for transporting several anti-inflammatory, anti-oxidant, and cytoprotective transcription factors from the nucleus to the cytoplasm^60,63,64^. Consequently, XPO1 inhibition may affect many inflammatory/immune pathways relevant to neurological diseases, including signaling cascades involving Nrf2, NF-ĸB, and FOXOs. Low doses of SINE compounds modestly improved neuronal survival in control neurons and those expressing TDP43^*WT*^-EGFP, but at these doses we failed to detect a change in the distribution of XPO1 target substrates, including the NLS-mCherry-NES reporter and endogenous Hsc70. Although we cannot rule out the possibility that the distribution of other XPO1 cargos was affected, the inability of low dose SINE compounds to alter the nucleocytoplasmic distribution of the NLS-mCherry-NES reporter—which carries a strong NES, as evidenced by the nuclear exclusion of this construct at steady-state—suggests that the beneficial effects of SINE compounds may be separate from XPO1’s role in protein trafficking^70^. Moreover, high doses of SINE compounds that exhibited measurable effects on nuclear trafficking also elicited significant toxicity when applied to primary neurons, consistent with the conclusion that neuroprotection by SINE compounds is separable from their effects on nucleocytoplasmic shuttling.

The predicted TDP43 NES lies within residues 239-250, partially overlapping the second RNA recognition motif^29,30^. Based on homology to the hydrophobic linear sequence recognized by XPO1^32^, TDP43 was presumed to be a XPO1 substrate. Moreover, genetic disruption of the presumed TDP43 NES resulted in the formation of insoluble nuclear aggregates^29^ and reduced the toxicity of cytoplasmically-mislocalized TDP43^18^. However, TDP43 was not among the list of XPO1 target proteins identified by 2 independent proteomic screens^63,64^, and no data has previously demonstrated a clear change in TDP43 localization when this domain is deleted or disrupted. These observations, together with our data showing that XPO1 overexpression results in a modest increase in cytoplasmic TDP43^*WT*^–mApple, but XPO1 inhibition by SINE compounds and leptomycin B fail to affect TDP43^*WT*^-mApple localization, suggest that XPO1 is not the only exporter responsible for nuclear TDP43 egress. Supporting this interpretation, overexpression of XPO7 and NXF1 as well as XPO1 effectively facilitated nuclear TDP43^*WT*^-mApple export. Prior studies confirm that NXF1 physically interacts with TDP43^71^, consistent with its role in TDP43 nuclear export, but this is the first indication that TDP43 may be an XPO7 cargo protein. In contrast, XPO5, whose yeast homolog MSN5 was previously identified as a genetic modifier of TDP43 toxicity^71,72^, showed no effect on TDP43 localization or toxicity in our assays. In fact, among the 8 nuclear exporters tested, only XPO7 depletion significantly increased the mean TDP43^*WT*^-mApple NCR, albeit only by 1%. Little is known about XPO7 cargo proteins^73^, and investigation into a potential XPO7/TDP43 interaction represents a promising future avenue of investigation.

Disrupted nucleocytoplasmic transport has emerged as a common underlying theme in age-dependent neurodegenerative diseases. Amyloid, mutant huntingtin^27,55^, mutant TDP43^18^ and mutant FUS^74^ have all been associated with abnormalities in nucleocytoplasmic transport. In addition, the most common mutation responsible for ALS and FTD — hexanucleotide expansions in the first intron of *C9orf72*^11,12^ — also impairs nucleocytoplasmic transport^22–25,75^. While XPO1 knockdown enhanced toxicity in some models of mutant *C9orf72*-related ALS/FTD^24,25^, XPO1 inhibition by SINE compounds mitigated toxicity in others^22^. In light of our data demonstrating a mild neuroprotective effect of SINE compounds independent of their effects on nucleocytoplasmic transport, these observations provide additional evidence that SINE compounds likely act through alternative mechanisms to relieve toxicity in disease models. Consequently, future *in vitro* and *in vivo* studies are warranted to more closely examine the effect of SINE compounds in models of ALS, FTD and related disorders. Identifying the downstream effectors of SINE compound-mediated neuroprotection is an important next step towards the goal of developing more potent agents with a wider therapeutic index. Finally, our finding that multiple exporter proteins mediate TDP43 nuclear egress underscores the need for further characterization of novel transport motifs within TDP43; this information is crucial for precision targeting of TDP43 nucleocytoplasmic transport as a therapeutic strategy in ALS and FTD.

## Materials and Methods

### Plasmid construction

All constructs expressed in primary neurons were cloned into pGW1, including TDP43^WT^-EGFP, TDP43^A315T^-EGFP, TDP43^WT^-mApple and TDP43^A315T^-mApple^21,38^, or Htt96Q-EGFP^37^. Coding sequences for XPO5, XPO1/CRM1 and NXF1 were PCR amplified from Addgene plasmids #58331 (Gift of Matthew Wood), #17647 (Gift of Xin Wang), and DNASU plasmid HsCD00442075, respectively, and cloned into a pGW1 vector containing EGFP-2A, allowing for equimolar expression of the survival marker EGFP and the untagged exporter. The XPO7 coding sequence was amplified by PCR, adding kozak and stop codon sequences, from DNASU plasmid HsCD00640055, and inserted into PGW1. The NLS-mCherry-NES reporter (pDN160) was a gift from Barbara Di Ventura & Roland Eils (Addgene plasmid # 72660).

### Adeno-associated virus preparation

The transgene expression cassette included AAV2 terminal repeats, the hybrid cytomegalovirus/chicken beta-actin promoter, human wild-type TDP43, and the bovine growth hormone polyadenylation sequence^76^. Helper and AAV9 capsid plasmids were from the University of Pennsylvania^77^. The expression cassette was packaged into recombinant AAV9 as previously described^76^. Viral stocks were sterilized using a Millex-GV syringe filter (Millipore), aliquoted, and frozen. Viral genome copies were titered using a dot-blot assay.

### Ethics statement

All vertebrate animal work was approved by the Committee on the Use and Care of Animals (UCUCA) at the University of Michigan, and the Louisiana State University Health Sciences Center at Shreveport’s Animal Care and Use Committee (ACUC). All experiments were performed in accordance with UCUCA and ACUC guidelines. Rats (*Rattus norvegicus*) used for primary neuron collection were housed singly in chambers equipped with environmental enrichment. Rats used for *in vivo* studies were housed with the dam until weaning at three weeks of age. Thereafter, they were housed in pairs by gender. All studies were designed to minimize animal use. Rats were cared for by the Unit for Laboratory Animal Medicine at the University of Michigan or veterinary specialists at Louisiana State University; all individuals were trained and approved in the care and long-term maintenance of rodent colonies, in accordance with the NIH-supported Guide for the Care and Use of Laboratory Animals. All personnel handling the rats and administering euthanasia were properly trained in accordance with the UM Policy for Education and Training of Animal Care and Use Personnel. Euthanasia was fully consistent with the recommendations of the Guidelines on Euthanasia of the American Veterinary Medical Association.

### *In vivo* animal studies

40 Sprague-Dawley rats (Harlan, Indianapolis, IN) of both genders from 6 different litters were used for these studies. Animals received temporal vein injections on post-natal day 1 and were monitored for 8 weeks. Animals were either injected with AAV9 TDP43 at a dose of 1 × 10^12^ vector genomes (vg; N = 23) or left uninjected (N = 17). KPT-350 was dissolved in a combination of poloxamer pluronic F-68 (6 mg ml^−1^) and plasdone PVP K-29/32 (6 mg ml^−1^) in sterile water (vehicle). For the neonatal rats, dosing began at post-natal day 2. Neonatal rats were given KPT-350 or vehicle (N = 19) twice per week via a small rubber tube attached to a 1-ml syringe. Two doses of KPT-350 were used, 7.5 mg kg^−1^ (N = 13) and 10 mg kg^−1^ (N = 8).

Performance on the rotarod was measured at 4 and 8 weeks as described (Wang *et al*.^57^). The rats’ grip strength was studied at 3 weeks using a hang test. Here, rats were placed on a cage cover with a metal grate and then held above a cage with ample bedding. The grate was turned to a vertical position and the amount of time the rat remained on the grate was timed (latency to fall). Trials were capped at 60 seconds. Three trials per rat were conducted and averaged.

### Cell culture and transfection

Rodent primary cortical neurons were prepared as described in^18^. Briefly, neurons dissected from embryonic day 20-21 Long Evans rat pups were cultured at a density of 0.6 × 10^6^ cells ml^-1^ in 96 well plates coated with laminin (Corning) and D-polylysine (Millipore). Euthanasia for these experiments is entirely consistent with the recommendations of the Guidelines on Euthanasia of the American Veterinary Medical Association. Primary neurons were transfected with plasmids and/or siRNA (obtained from Dharmacon) 4 days after plating using Lipofectamine 2000 (Invitrogen)^21^. SINE compounds KPT-335 and KPT-350 (Karyopharm Therapeutics), Leptomycin B (Cayman Chemicals) or vehicle (DMSO, Sigma) were added 24 h after transfection, immediately following the first imaging run. In experiments measuring subcellular localization of fluorescent proteins, the nucleus was identified using cell-permeable nuclear markers (Hoechst 33342, Sigma).

HEK293 cells were passaged every 3-4 days (~90% confluence) using trypsin. For transfection, HEK293 cells were passaged 1:10 and transfected 24 h later using Lipofectamine2000 (Invitrogen), according to the manufacturer’s protocol, and cell-permeable nuclear marker DRAQ5 (Thermo Fisher) added post-transfection.

### Immunoblot and Immunocytochemistry

For subcellular fractionation, primary cortical neurons from embryonic day 20-21 rats were prepared as described above, and treated with drug or vehicle 6 days after plating. Cells were harvested 7 h after treatment in resuspension buffer (10 mM Tris (pH 7.4), 10 mM NaCl, 3 mM MgCl_2_) supplemented with 0.6% Igepal CA-30 (Sigma) to liberate the cytoplasmic fraction, and then in RIPA buffer to lyse the nuclear compartment, following a trypan blue assay for nuclear integrity. Protein content determined by the BCA assay (Pierce) using a Nanodrop 2000. Samples normalized for protein content were run on 15% SDS polyacrylamide gels. Primary antibodies against TDP43 (Barmada *et al*.^21^), Hsc70 (1:1000, Santa Cruz SC-7298), GAPDH (1:1000, Millipore MAB374), and Histone H2B (1:250 Novus Biologicals 50347) were detected using IR dye conjugated secondary antibodies (LI-COR Biosciences), imaged on a LI-COR Odyssey, and quantified using ImageStudioLite (LI-COR Biosciences). Plots were generated and analyzed using GraphPad Prism.

For immunoblots of animal brain, fresh tissues were collected and immediately subjected to nuclear and cytosolic fractionation using a fractionation kit (NE-PER Nuclear and Cytoplasmic Fractionation Kit, Pierce, Rockford, IL). Fractionation followed manufacturer’s protocol. Protein content determined by the Bio-Rad Protein Assay Dye. Samples normalized for protein content were run on 4-15% SDS polyacrylamide gels. Primary antibodies include anti-TDP43 (1:2000, ProteinTech 107282-2-AP), anti-human TDP43 (1:1500, Abnova H00023435-M01), anti-GAPDH (1:2000, Ambion AM4300), and anti-lamin B2 (1:2000, Millipore MAB3536). Secondary antibodies were from Santa Cruz Biotechnologies (Dallas, Tx), and were used at a dilution of 1:10000. Chemiluminescence reagents were purchased from Amersham (Buckinghamshire, UK).

For detection of XPO7 by immunocytochemistry, primary cortical neurons from embryonic day 20-21 rats were prepared as described above, then transfected with plasmids encoding EGFP and XPO7 4 days after plating. Cells were fixed with 4% paraformaldehyde, permeabilized with 0.1% Triton-X-100, and treated with 10 mM glycine before blocking in PBS containing 0.1% Triton, 2% FCS and 3% BSA, for 1 h at room temperature (RT). Detection was accomplished by incubating with anti-XPO7 primary antibodies (diluted 1:500 in blocking solution, (Proteintech, 12980-1-AP) overnight at 4 °C. Samples were rinsed 3 times in PBS, incubated with Cy3 labeled anti-rabbit secondary antibodies (1:250 in blocking solution; Millipore, AP132C) for 1 h at RT, then rinsed 3 more times in PBS containing a 1:1000 dilution of Hoechst 33258 nuclear dye (Invitrogen H3569) prior to imaging.

### Fluorescence microscopy

Primary cortical neurons and HEK293 cells were imaged using an automated microscopy platform described previously^18,21,38^. Briefly, images were obtained with an inverted Nikon TiE-2000 microscope equipped with a Nikon PerfectFocus 3 system, a high-numerical aperture 20x objective lens, Semrock BrightLine filter sets, and a 16-bit Andor iXon digital camera with an electron multiplied ultra-cooled charge-coupled device. The Lambda XL Xenon lamp provided broad-spectrum illumination via a 5-mm liquid light guide. Stage movements were accomplished using an ASI 2000 stage with rotary encoders in the x- and y-axes. The microscope and associated components were encased in a climate-controlled environmental chamber that we built specifically for this purpose. The illumination, filter wheels, focusing, stage movements, and image acquisitions were fully automated and coordinated with publicly available (ImageJ, µManager) software.

### Image analysis

Neuronal survival was assessed in an unbiased and blinded manner using novel code written in Python. Twenty-five images per well of a 96 well plate were stitched together to cover a larger surface area, background fluorescence subtracted from the stitched images, and images from each timepoint assembled into a time series or stack. Individual neuronal soma and processes were segmented based on morphological characteristics, and time of death recorded as the last time a neuron was confirmed to be alive (left censoring). Cumulative and instantaneous risk of death plots were calculated from this information using the survival analysis package in R. Statistical comparisons (Cox proportional hazards analysis and the log-rank test) were also accomplished in R.

Regions of interest (ROIs) identified by blinded observers (Figs 5 and 6, S6) or by automated analysis (Figs 7 and 8, S6) corresponding to the cell body and nucleus were used to calculate the mean intensity of fluorescently-labeled proteins within specific subcellular compartments. Expression of EGFP in these experiments was necessary to outline the cell body and draw ROIs around the cytoplasm of transfected cells. For automated analysis, nuclei were identified and partitioned based upon their intensity via Baysian Gaussian Mixture modelling. Cells were removed from the analysis if the nuclear intensity distribution suggested pyknosis, or if the soma-centroid estimate was not contained within the innermost contiguous group of pixels. Data was compiled using R, and scatter plots and graphs depicting changes in protein localization were created using GraphPad Prism.

### Verification of siRNA-mediated exporter knockdown

Rat PC12 cells maintained in culture were transiently transfected with 25 nM Dharmacon smartpool siRNA constructs, and harvested 48 hours post transfection, using the Qiagen RNAeasy kit, following the manufacturers protocol. Transcript abundance was determined by quantitative (q)PCR using Power SYBR Green (Applied Biosystems/Thermofisher). 4 ul of a 20 ul cDNA reaction (reverse transcribed from 1 ug of RNA with the Bio-Rad iScript kit) for each sample was used as template with the following primers:

GAPDH  FWD: AGTGCCAGCCTCGTCTCATA 

REV: GGTAACCAGGCGTCCGATAC

XPO1   FWD: TTGTGACAGACACCTCACATAC   

REV: TAGCGGTGTGCTTATCTTTCC

CSE1L  FWD: GAAGCTGCTGGCTGTTAGTA    

REV: GGGTTAGCTTTGCAGGTTATTC

XPOT   FWD: CCGGGATGTGGTTCATACAT   

REV: CACTGTTCCCTCATGGTATCTT

XPO4     FWD: GTGAGCTCTTCTCCTCCTTTG    

REV: ATGTACACCATGTCGTCCTTATC

XPO5:   FWD: CTGGAAGCCCTCAAGTTTTG    

REV: CTTCTCCAATCGAGACATGC

XPO6    FWD: CTCAACTCTGTTCCCAGTTCTT     

REV: TTCTCCGCTGTGATGTTCAG

XPO7    FWD: CAGACGTCACGAGGTTCTTAC     

REV: GGGTGGTGTCTGCTTGATTA

NXF1    FWD: GTGGCTTGACAGAAACCCCA     

REV: ATGGCCATCCAGGCGTAATAA

GAPDH was used as an internal control for each sample. Knockdown efficiency was gauged by scaling all relative transcript abundances to the value obtained in cells transfected with scrambled siRNA.

### Data availability statement

The materials and datasets generated during and/or analyzed during the current study are available from the corresponding author on reasonable request.

## Electronic supplementary material

Supplementary information
