# Supplementary material for: TDP43 nuclear export and neurodegeneration in models of amyotrophic lateral sclerosis and frontotemporal dementia
Source: Sci Rep. 2018 Mar 15;8:4606. doi: 10.1038/s41598-018-22858-w (PMC5854632; doi:10.1038/s41598-018-22858-w)
Supplement: Supplementary file 1 — Supplementary information [file 41598_2018_22858_MOESM1_ESM.pdf]

Supplementary Information

TDP43 nuclear export and neurodegeneration in models of amyotrophic lateral sclerosis and frontotemporal dementia

Hilary C Archbold[1], Kasey L Jackson[2], Ayush Arora[1], Kaitlin Weskamp[1,3], Elizabeth M-H Tank[1], Xingli Li[1], Roberto Miguez[1], Robert D Dayton[2], Sharon Tamir[4], Ronald L Klein[2], and Sami J Barmada* [1,3,5]

[1]Department of Neurology, University of Michigan, Ann Arbor, MI; [2]Department of Pharmacology, Toxicology & Neuroscience, Louisiana State University Health Sciences Center, Shreveport, LA; [3]Neuroscience Graduate Program, University of Michigan, Ann Arbor, MI; [4]Karyopharm Therapeutics, Newton, MA; [5]Cellular & Molecular Biology Program, University of Michigan, Ann Arbor, MI

* To whom correspondence should be addressed: Sami Barmada, 109 Zina Pitcher Place, 5015 Alfred A Taubman Biological Sciences Building, Ann Arbor, MI 48109. Tel: 734-763-2624. Fax: 734-764-6493. Email: sbarmada@umich.edu

Supplemental figure legends

Supplemental Figure 1: Acute toxicity of SINE compounds when administered with transfection skews survival analyses. The total (**a**) and mean (**b**) number of neurons identified 24 h after transfection and treatment with KPT-335 exhibits an inverse relationship with the drug dose. Treatment with KPT-350 at the time of transfection produced a similar, although not statistically significant effect, in cells expressing mApple, TDP43^{WT} or TDP43^{A315T}-mApple (**c**, mean # fluorescent cells). This effect decreases the slope of the risk of death, particularly with overexpression of TDP43^{WT} or TDP43^{A315T}. (**e-h**) Primary neurons were transfected with EGFP or Htt^{96Q}-EGFP, and KPT-335 or vehicle control added at the time of transfection. KPT-335 treatment consistently reduced the mean number of living cells detected 24 or 48 h after application (**e**), although the effect was more pronounced at 48 h. When survival analysis was initiated 24 h after transfection and drug addition, 50 nM KPT-335 significantly increased the risk of death for neurons expressing Htt^{96Q}-EGFP (**h**, HR = 1.34), with acute toxicity from drug addition peaking between 24 and 48 h post transfection (**f,** pink arrows in cumulative (left) and instantaneous (right) hazard plots). The instantaneous risk of death decreases over time (**f,** pink arrowhead). When survival analysis was initiated 48 h post transfection (**g**), after acute toxicity from drug addition has waned (**g**, pale pink arrows, in cumulative (left) and instantaneous (right) hazard plots), an apparent neuroprotective effect for KPT-335 emerges (**g,** pink arrowheads), and the HR for KPT-335 treatment decreases from 1.34 to 0.82 (**h**). Values in (**a, d**) and (**b, e**) were pooled or averaged, respectively, from 2 or 4 biological replicates. Survival plots in (**g**) and (**h**) were generated from 8 technical replicates in each biological replicate. Plots (**b**), (**c**) and (**e**) show mean ± SD; *p<0.05, **p<0.01, ***p<0.001, 2-way ANOVA with Dunnett's multiple comparison.

Supplemental Figure 2: TDP-43 overexpression *in vivo*. Brains from uninjected and AAV9 TDP43-injected rats were collected and analyzed by SDS-PAGE and Western blotting. (**a**) Full- length blot detected with anti-GAPDH antibodies (1:2000, Ambion AM4300). (**b**) Full-length blot detected with anti-TDP43 antibodies (1:1500, Abnova H00023435-M01). Arrow indicates overexpressed human TDP43. * denotes non-specific band.

Supplemental Figure 3: Effects of KPT-350 administration in rats. (**a**) Rotarod testing at 8 weeks demonstrated prominent motor deficits in the TDP43-expressing animals. Treatment with KPT-350 at 7.5 or 10 mg kg^{-1} (grey and black bars, respectively) failed to rescue these deficits. n, number of rats, mean ± s.e.m, ns, not significant. ***, p < 0.001 one-way ANOVA with Bonferroni correction. KPT-350 treatment (orange and red lines) significantly reduced weight gain in both male (**b**) and female rats (**c**) (ANOVA, p < 0.001). TDP43 expression (blue line) significantly reduced weight gain only in male rats (p < 0.05). There was no statistical interaction between KPT-350 and TDP43 expression in males or female

rats.

Supplemental Figure 4: The annotated bipartite NES of TDP43 is surface exposed. (**a**) FirstGlance in Jmol was used to view the NMR structure of TDP43 RNA recognition regions (RRM) 1 and 2 in complex with RNA (from Lukavsky *et al.*, 2013). The residues that make up the bipartite NES at amino acid positions 239-243 and 248-250 (239-**IAQSL**CGED**LII**-250) are highlighted in yellow. (**b**) In the surface diagram, part of the annotated NES (residues 248-250) appears to be buried within the cleft between RRM1 and 2, while resides 239-243 are more exposed, suggesting that they could serve as recognition sites for export proteins or adapter molecules.

Supplemental Figure 5: SINE compounds produce no detectable change in TDP43 localization. (**a**) Rodent primary cortical neurons were treated with vehicle (DMSO) or SINE compounds at the indicated dose, then subject to subcellular fractionation and Western blotting using antibodies against Hsc70, an XPO1 substrate, and TDP43. The histone H2B was used as a marker for the nuclear fraction (NF), while GAPDH served as a marker of the cytoplasmic fraction (CF). (**b-c**) Quantification of the data shown in (**a**), demonstrating no change in the subcellular distribution of TDP43 or Hsc70 upon treatment with low-dose SINE compounds (mean ± s.d). Results were pooled from 3 independent experiments, performed in duplicate. (**d**) Subcellular fractionation of 8-week old brains from AAV9 TDP43-transduced rats treated with 7.5 mg kg^{-1} KPT-350 or vehicle. The cytoplasmic fraction (top) was marked by GAPDH, and the nuclear fraction (bottom) by lamin B2. (**e**) Quantification of data shown in (**d**), showing no significant difference in the nuclear/cytoplasmic ratio of TDP43 with KPT-350 treatment (mean ± s.d).

Supplemental Figure 6: Comparison of manual and automated nuclear fractionation methods. Blinded observers used Fiji to select regions of interest (ROIs) around the cell body (**a**, left panel, marked by diffuse mApple or, in this case, EGFP fluorescence) and nucleus (**a**, middle panel, stained with Hoechst dye). Area and integrated signal density for each ROI were measured within a third channel (**a**, right panel), here representing $TDP43^{WT}$-mApple, and the NCR calculated from the mean fluorescence intensity in each cellular compartment (plotted in **f**). The automated system uses a similar process, although it consistently selects more conservative cytoplasmic ROIs to avoid overlapping ROIs from neighboring neurons (**b**). Additionally, the perinuclear space is excluded from the analysis to prevent fluorescence bleedthrough from affecting cytoplasmic measurements. The automated system calculates a nuclear to cytoplasmic ratio (NCR; **h**) for each cell, representing the mean fluorescence intensity within the nuclear area (defined as the space within the green bounding ellipse, middle panel in **b**), divided by the mean fluorescence intensity within the cytoplasmic area (defined as the space between the blue and red bounded areas, generated by Bayesian Gaussian mixture modeling). If the nuclei are deemed pyknotic (**c**) or the soma-centroid estimate is not contained within the innermost contiguous group of pixels (**d**), cells

were excluded from the analysis. In some cases (**e**), overlapping nuclei from non-transfected cells triggered exclusion of the ROI. The relative differences between NCR for each condition were consistent as determined by the manual and machine-based analyses (compare **f** and **h**). The log-transformed NCR values generated by manual and machine counting are shown in panels (**g)** and (**i**), respectively. A single 96 well plate divided into 12 groups (8 wells per column) was used as a test set for the comparison.

Supplemental Figure 7: siRNA depletion of exporters results in approximately 40-60% reduction of targeted RNAs. Rat PC12 cells were transfected with 25 nM Dharmacon smartpool siRNA targeting the indicated exporter. 48 h after transfection, RNA was purified from each condition, and transcript abundance determined by qPCR. In each case, GAPDH was used as an internal control, and relative mRNA abundance for the tested exporter when treated with scrambled siRNA set to 1. The depicted average abundance values were calculated from 3 biological replicates, containing 3 technical replicates each (mean ± s.d). *$p<0.001$, one-way ANOVA with Dunnett's correction.

Supplemental Figure 8: Expression of untagged export proteins. Primary rodent cortical neurons were transfected with empty vector (EV, **a**) or untagged human XPO7 (**b**), and fixed after 24 h. Elevated expression of human XPO7, as detected by immunocytochemistry (**b**), is notable in EGFP-expressing cells, consistent with effective co-transfection. The far right panels of (**a**) and (**b**) depict the relative abundance of XPO7 in transfected cells with a false color intensity heatmap. (**c-f**) Overexpression of the exporters XPO7 (**c, d**), XPO1, XPO5 or NXF1 (**e, f**) in rodent primary cortical neurons is significantly toxic as measured by automated survival analysis and Cox proportional hazards (**d, f**).

Supplemental figure 1

a

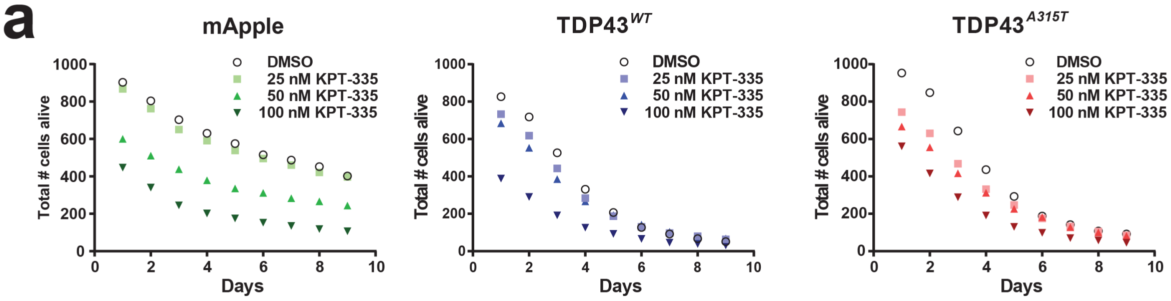

b

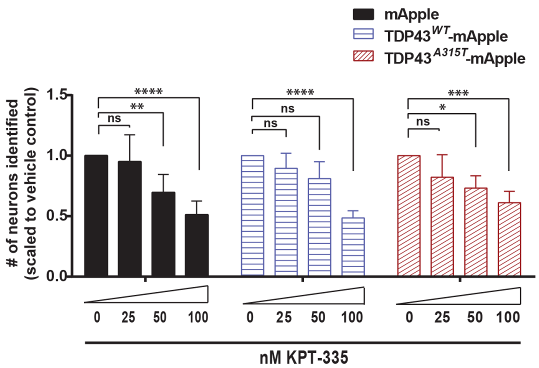

c

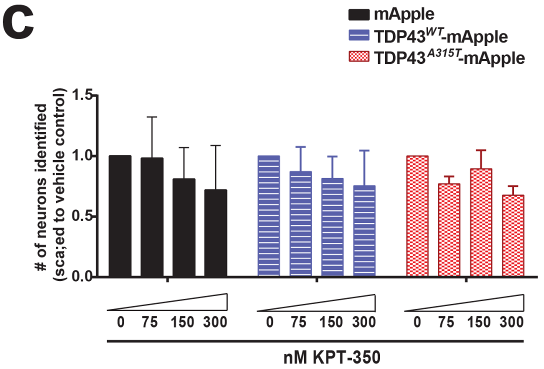

d

Neuron Identification Time	Replicate	# of cells			
		EGFP		Htt^{96Q}-EGFP	
		DMSO	50 nM KPT-335	DMSO	50 nM KPT-335
~ 24 hours post treatment	R1	164	219	245	248
	R2	385	220	593	309
~ 48 hours post treatment	R1	126	147	210	179
	R2	260	94	422	121
	R3	388	270	352	185
	R4	327	289	165	128

e

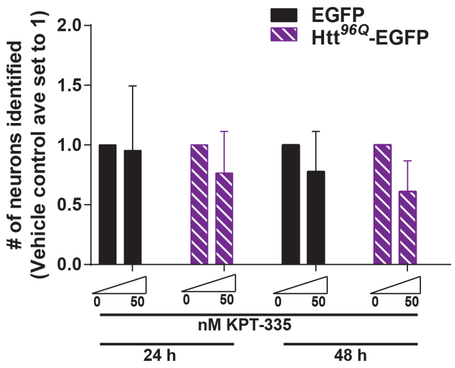

f

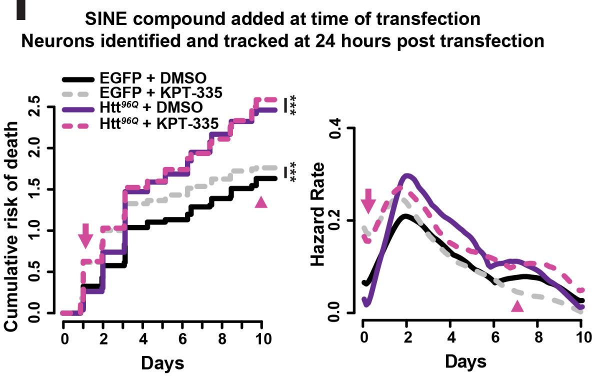

g

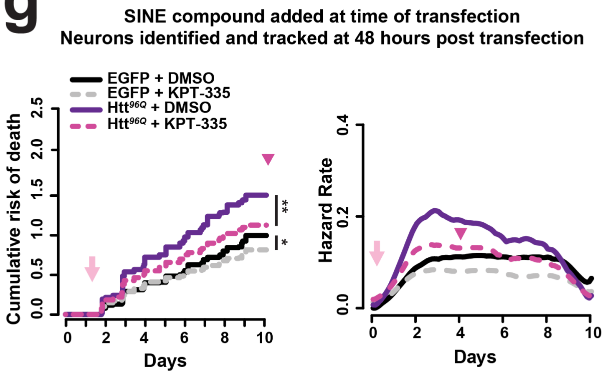

h

		Identify neurons 24 hours post transfection and drug addition				Identify neurons 48 hours post transfection and drug addition			
Treatment		#	HR	p	95% CI	#	HR	p	95% CI
EGFP	DMSO	594	reference			1101	reference		
	KPT-335	439	1.38	<0.001	1.20-1.59	800	0.86	<0.05	0.76-0.98
Htt^{96Q}-EGFP	DMSO	838	1.31	<0.001	1.17-1.48	1149	1.53	<0.001	1.38-1.69
	KPT-335	557	1.71	<0.001	1.50-1.95	613	1.21	<0.01	1.07-1.38
Htt^{96Q}-EGFP	DMSO	838	reference			1149	reference		
	KPT-335	557	1.34	<0.001	1.20-1.51	613	0.82	<0.01	0.73-0.93

Supplemental figure 2

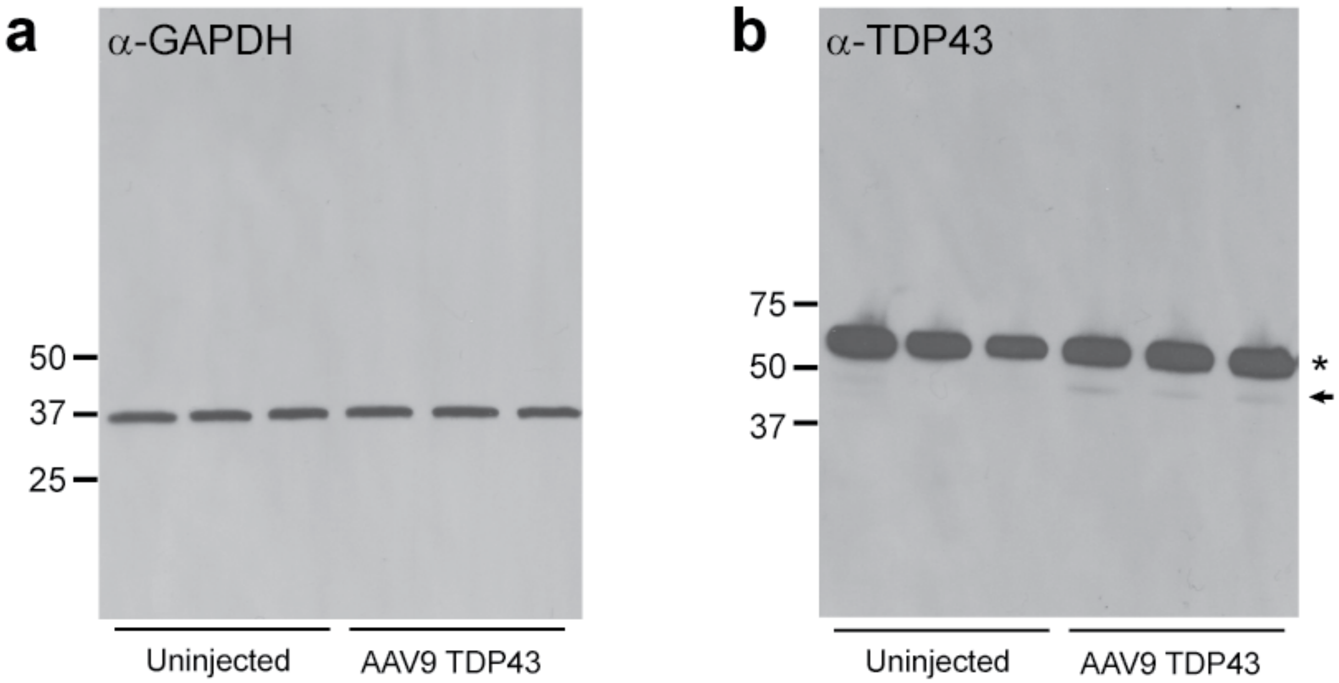

Supplemental figure 3

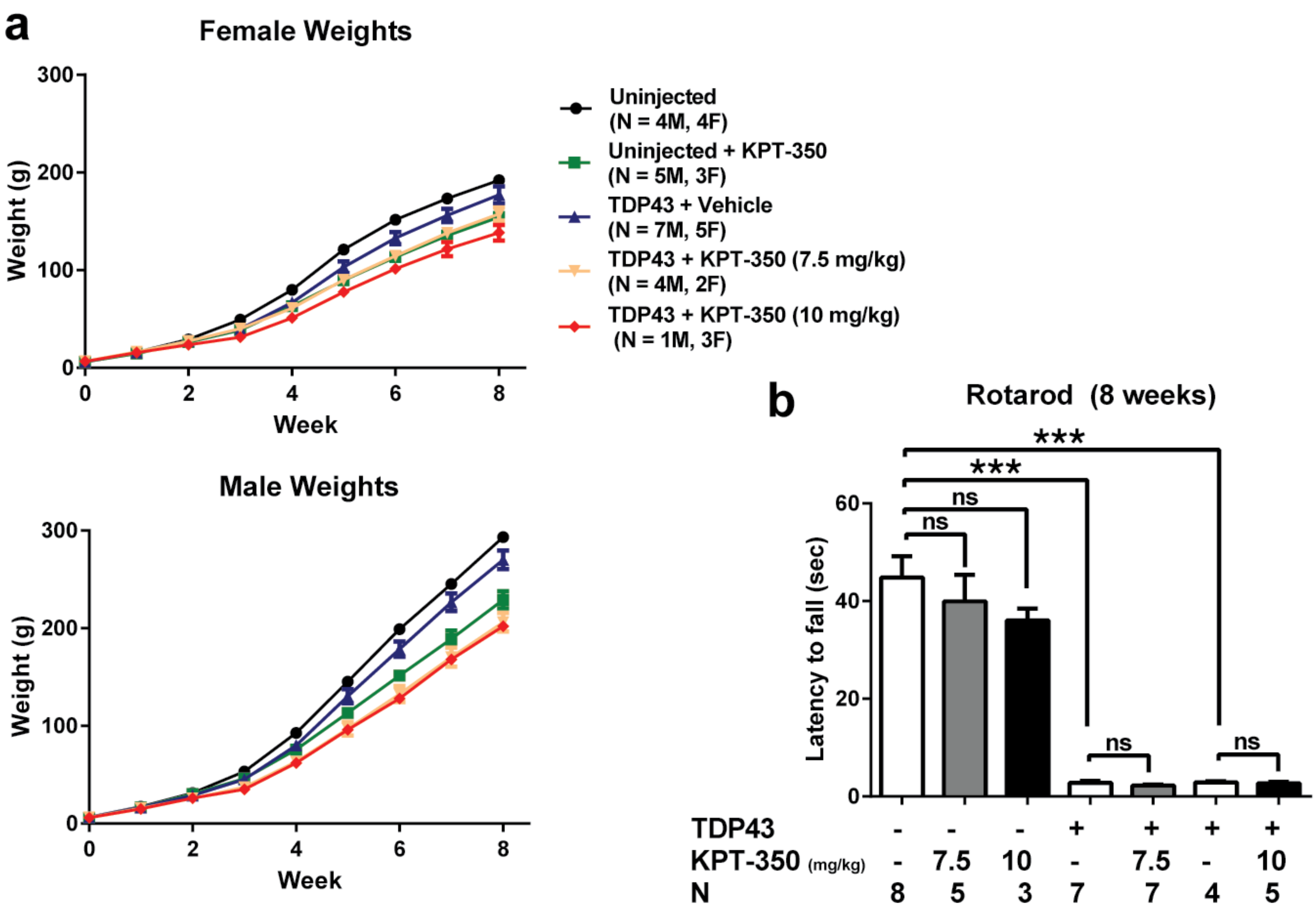

Supplemental figure 4

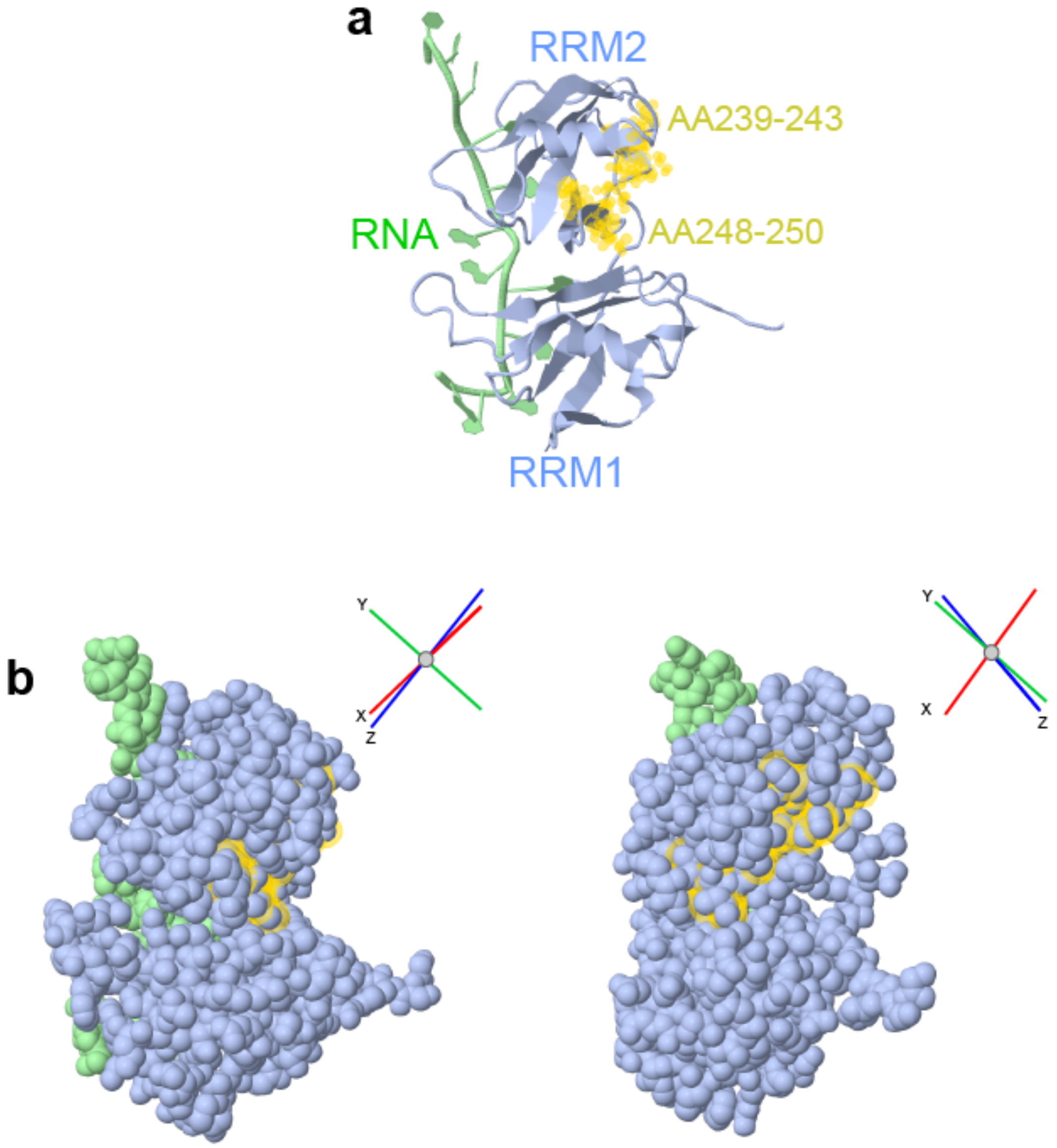

Supplemental figure 5

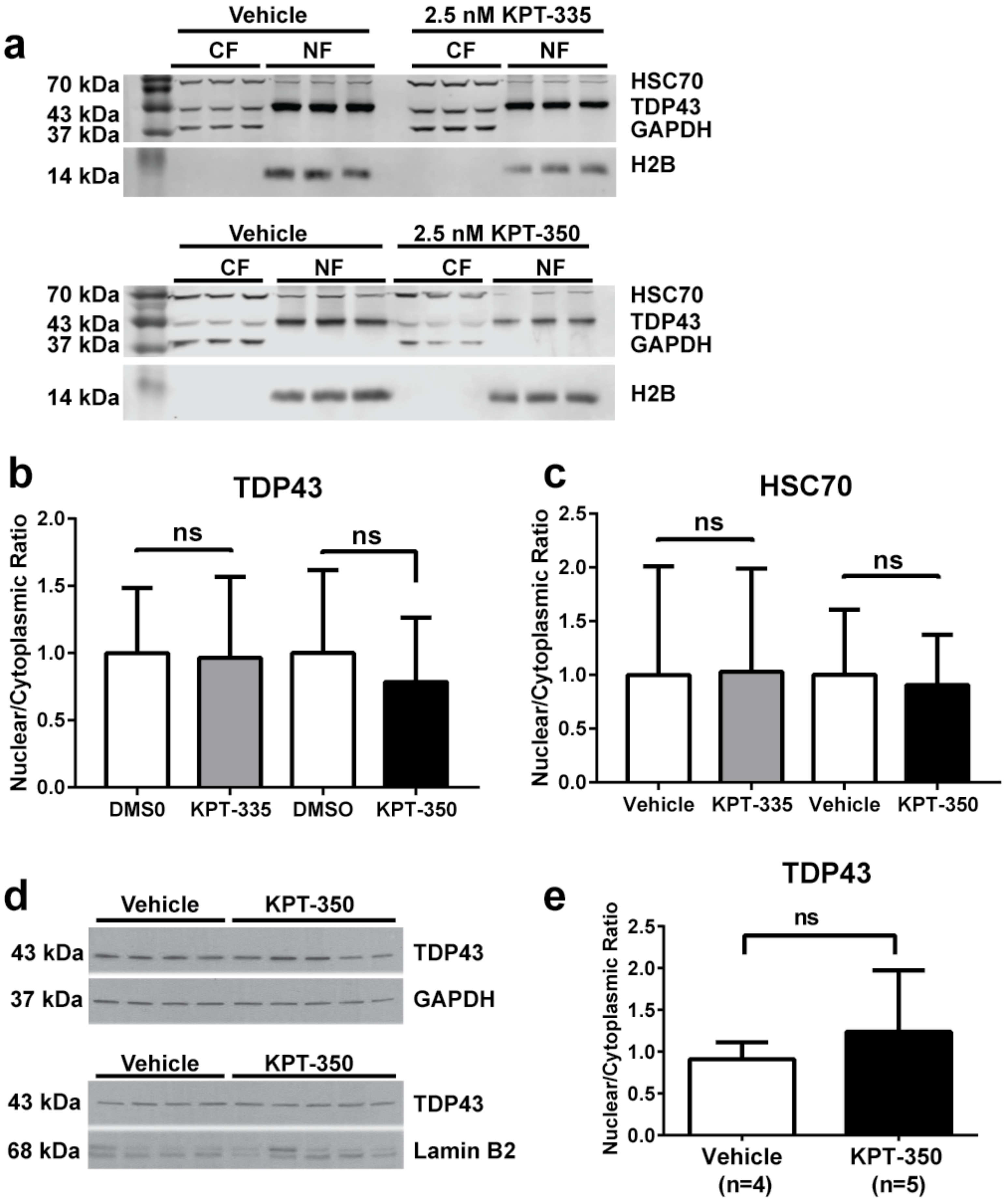

Supplemental figure 6

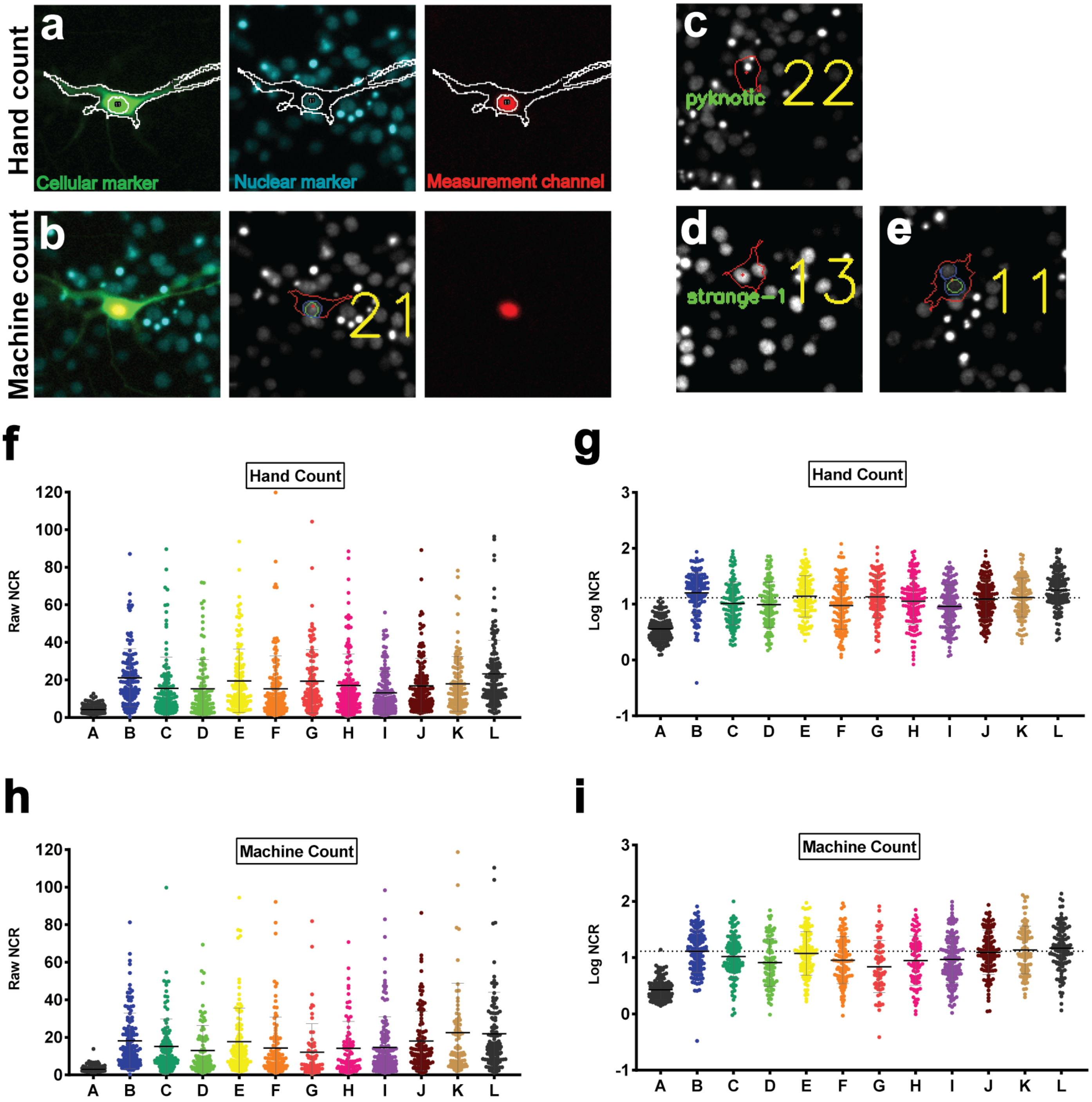

Supplemental figure 7

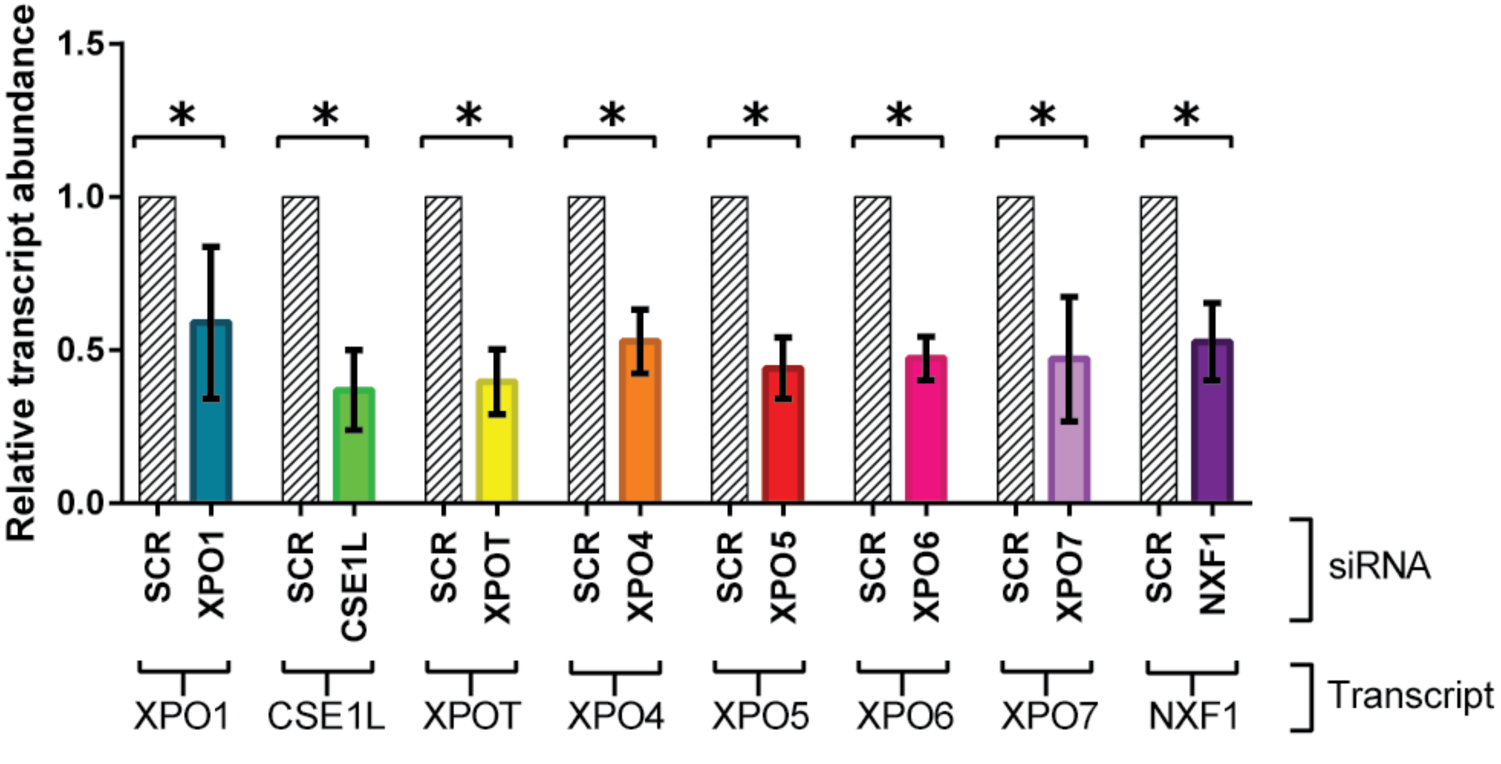

Supplemental figure 8

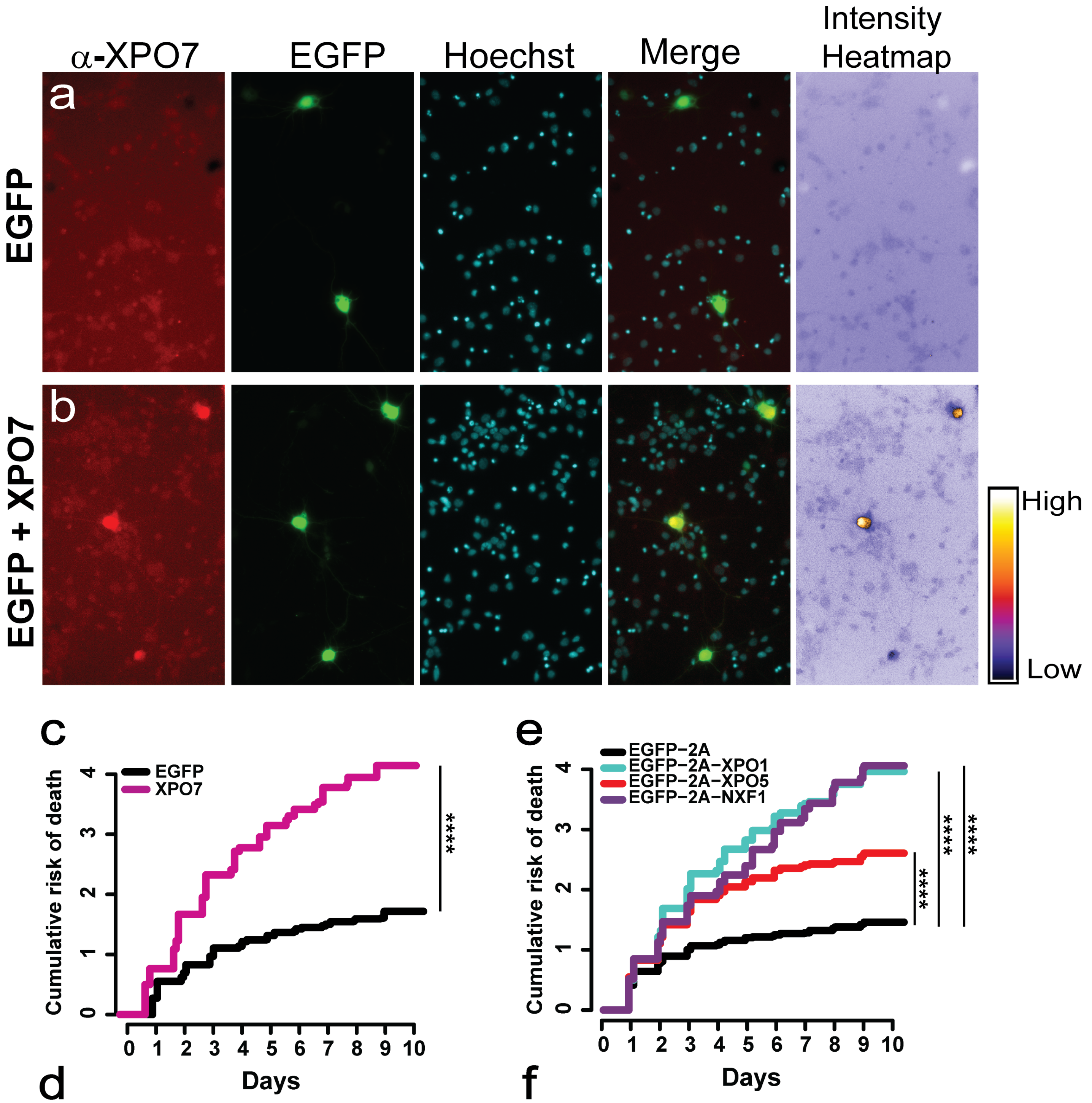

d

Treatment	#	HR	p	95% CI	LR p
EGFP	654	reference			
XPO7	698	1.97	<0.001	1.75-2.21	<0.001

f

Treatment	#	HR	p	95% CI	LR p
EGFP-2A	1248	reference			
EGFP-2A-XPO1	891	2.02	<0.001	1.83-2.22	<0.001
EGFP-2A-XPO5	814	1.70	<0.001	1.54-1.88	<0.001
EGFP-2A-NXF1	1236	1.91	<0.001	1.75-2.08	<0.001
